# Influence of growth stage on the chemical composition, antimicrobial, and antioxidant potential of *Cymbopogon martinii* (Roxb.) Wats. essential oil

**DOI:** 10.3389/fpls.2025.1660363

**Published:** 2025-09-12

**Authors:** Priyankaraj Sonigra, Mukesh Meena

**Affiliations:** Laboratory of Phytopathology and Microbial Biotechnology, Department of Botany, Mohanlal Sukhadia University, Udaipur, Rajasthan, India

**Keywords:** *Cymbopogon martinii*, growth stages, essential oils, post-harvest pathogen, antimicrobial activity, antioxidant activity

## Abstract

**Introduction:**

*Cymbopogon martinii* (Roxb.) Wats. essential oil (CMEO) exhibits significant variation in composition and bioactivity across different growth stages. Understanding these changes is crucial for optimizing its therapeutic and industrial applications.

**Methods:**

CMEO was extracted at the vegetative, reproductive, and post-reproductive stages using hydro-distillation. Chemical composition was analyzed by GC-MS. Antimicrobial activity was assessed using disc diffusion and direct contact assays, while antioxidant potential was evaluated through DPPH, ABTS, and β-carotene bleaching assays. Correlation analysis was performed to link major bioactive compounds with biological activities.

**Results:**

A total of 59 compounds were identified, with the reproductive stage showing the highest diversity (49 compounds, 97.86%) and oil yield. Major compounds varied across stages: carveol (20.87%), trans-p-mentha-1(7),8-dien-2-ol (12.9%), and D-limonene (6.2%) dominated the vegetative phase; cis-piperitol (15.27%), cis-p-mentha-1(7),8-dien-2-ol (15.52%), and carvone (3.31%) were abundant in the reproductive phase; while the post-reproductive phase was rich in trans-p-mentha-1(7),8-dien-2-ol (19.58%) and carveol (11.32%). Antibacterial and antifungal activities were highest during the reproductive stage, particularly against *Staphylococcus aureus* and *Alternaria alstroemeriae*. Antioxidant potential peaked during the post-reproductive phase, with the lowest IC_50_ values.

**Discussion:**

Correlation analysis revealed negative associations between key bioactive compounds (e.g., carvone, D-limonene, α-methylcinnamaldehyde, and (S)-perillyl alcohol) and microbial/oxidative inhibition thresholds, confirming their contribution to CMEO bioactivity. These findings highlight the critical role of harvest timing in maximizing the chemical richness, antimicrobial efficacy, and antioxidant potential of CMEO.

## Graphical Abstract

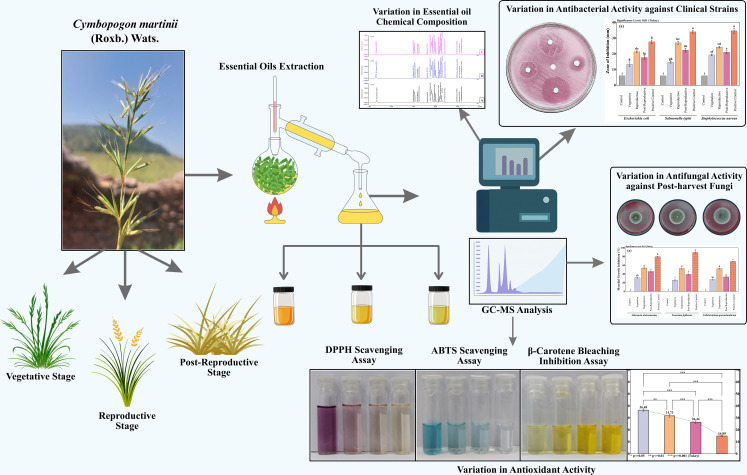

## 1 Introduction

*Cymbopogon martinii* (Roxb.) Wats., commonly known as palmarosa, is a perennial aromatic grass of the Poaceae family, cultivated primarily for its essential oil (EO), which holds high economic and industrial value in the pharmaceutical, cosmetic, and food sectors. It has gained significant economic importance due to its essential oil, which is rich in geraniol and other oxygenated monoterpenes. Globally, the palmarosa oil market was valued at approximately USD 120 million in 2023 and is projected to reach around USD 220 million by 2032, growing at a compound annual growth rate (CAGR) of ~6.5% (Dataintelo, 2023). India is the leading producer, accounting for nearly 80% of the global output, with an estimated production volume of approximately 2,000 metric tons per year (Market Report Analytics, 2023). Export analysis of Indian palmarosa oil between 2000 and 2020 revealed a CAGR of 22.3% in volume and 39.2% in export value, reflecting robust international demand in aromatherapy, perfumery, cosmetics, and natural preservatives (Choudhri et al., 2022). These trends underline the industrial and therapeutic relevance of optimizing harvest timing and essential oil composition, as investigated in the present study. Native to the Indian subcontinent and parts of Southeast Asia, *C. martinii* is one of approximately 140 species within the *Cymbopogon* genus, many of which are renowned for their rich essential oil content and broad biogeographical distribution across Africa, Asia, Australia, and the Americas (Jagadish Chandra, 1975; Prashara et al., 2003; Rao et al., 2005). Essential oils are volatile, aromatic plant metabolites stored in specialized secretory tissues and are widely recognized for their functional roles in plant defense and communication. Their bioactive properties, particularly antimicrobial and antioxidant activities, are increasingly valued as natural alternatives to synthetic preservatives in food systems and other applications (Rastogi et al., 2001; Mkaddem Mounira, 2024). These properties are largely attributed to diverse classes of phytochemicals, including monoterpenes, sesquiterpenes, aldehydes, and phenolic compounds.

The essential oil of *C. martinii* (CMEO) is rich in biologically active constituents such as geraniol, geranyl acetate, linalool, farnesol, caryophyllene, and nerolidol, which have demonstrated antimicrobial, antioxidant, insecticidal, and anti-inflammatory properties (Mallavarapu et al., 1998; Raina et al., 2003; Kakaraparthi et al., 2015; Liu et al., 2017; Chellappandian et al., 2018; Sharma et al., 2023; Tit and Bungau, 2023). Prior research has established that, the chemical composition and efficacy of EOs can vary considerably based on several factors, including geographic location, climate, soil conditions, and notably, the plant’s developmental stage at the time of harvest (Moghaddam and Mehdizadeh, 2017; Chrysargyris et al., 2021; Jangi et al., 2022). Growth stage–related variation in EO composition is especially critical, as it can directly affect the biological activity and commercial value of the oil (Saeb and Gholamrezaee, 2012).

Although the essential oil (EO) of *Cymbopogon martinii* has been previously studied for its phytochemical composition and biological activities, most existing research has focused on a single growth stage, with limited exploration of compositional variation throughout the plant’s phenological development. Earlier studies often emphasized dominant compounds such as geraniol, without examining how the full metabolite profile and associated bioactivities shift over time. Moreover, few reports have integrated metabolomics-based tools to statistically associate specific volatile constituents with biofunctional outcomes. In contrast, the current study applies a stage-wise comparative approach combining GC-MS profiling with multivariate and correlation analysis to systematically assess how key metabolites correlate with antimicrobial and antioxidant activities. This integrated analysis framework offers a novel contribution to *C. martinii* metabolomics by uncovering stage-specific bioactive markers and *vs*orting evidence-based harvest strategies to maximize essential oil efficacy. To the best of our knowledge, such a comprehensive phytochemical–bioactivity correlation across growth stages has not yet been reported for this species.

## 2 Experimental methodology

### 2.1 Plant sample collection

The aerial parts of *C. martinii* were gathered at three distinct growth stages: (i) July (Vegetative stage), (ii) October (Reproductive stage), and (iii) January (Post-reproductive stage) from Kailashpuri, a hilly area in Udaipur, Rajasthan, India, across the years 2021 and 2022. The plant sample was submitted to the University of Rajasthan, Jaipur, India, for proper identification (Voucher number: RUBL21430). The collection site is located at 24° 44’ 46” N and 73° 43’ 20” E, with recorded environmental conditions: relative humidity between 33–72%, temperature ranging from 24–37°C, and precipitation varying from 76.8–235.6 mm.

### 2.2 Chemicals and reagents

All chemicals and reagents utilized in this study were of analytical grade, ensuring high purity for accurate and reliable results. Methanol (Merck, 34860, ≥99.9%) was employed as the solvent for gas chromatography-mass spectrometry (GC-MS) analysis. For the assessment of antioxidant activity, the following reagents were used: 2,2-diphenyl-1-picrylhydrazyl (DPPH) (Merck, 102839, ≥95%), ascorbic acid (Merck, A92902, ≥99%), ABTS (2,2’-azino-bis(3-ethylbenzothiazoline-6-sulfonic acid) diammonium salt) (Merck, A1888, ≥98%), potassium persulfate (Merck, 379824, ≥99%), β-Carotene (Merck, 22040, ≥97%), linoleic acid (Merck, L1376, ≥99%), and Tween 40 (Polysorbate 40) (Merck, P1504, ≥99%). These high-purity reagents were selected to ensure the reliability and consistency of the experimental results.

### 2.3 Volatile oil extraction and yield

The CMEOs was extracted following the method outlined by Rathore et al. (2022). Aerial parts of *C. martinii*, including leaves, stems, and flowers were collected at different intervals across the three growth stages: vegetative, reproductive, and post-reproductive. For each extraction, 500 g of fresh plant material was placed in a round-bottom flask (3 L capacity) with 1 L of water and subjected to hydro distillation for 4 hours using a Clevenger-type apparatus, suitable for extracting EOs lighter than water. The extracted oils were then dried over anhydrous sodium sulfate and stored in amber glass vials at 4°C until analysis. The essential oil yield at each growth stage was determined as a percentage of the fresh plant material weight, using the Equation (1).


(1) $$\text{Yield }\left(\%\right)=\frac{\text{Weight of Essential oil }\left(\text{g}\right)}{\text{Weight of Fresh Plant Material }\left(\text{g}\right)}\times 100$$

### 2.4 GC-MS analysis

The chemical profiling of the CMEOs was conducted with a Thermo Fisher Scientific gas chromatograph (TRACE™ 1300), coupled to a Thermo Fisher Scientific triple quadrupole mass spectrometer (TSQ 9000). The separation process utilized two columns: a TG-SQC front column (0.25 µm thickness, 0.25 mm ID, 15 m length) with 5% phenyl methyl polysiloxane and a TG-1MS back column (0.25 µm film thickness, 0.25 mm ID, 30 m length) made of 100% dimethyl polysiloxane. The oven temperature started at 60°C for an initial hold of 10 minutes. For analysis, 1 μL of diluted samples (1/100 in GC-MS grade methanol, v/v) was injected in split mode (split ratio 1/50) via an autosampler (AI 1310). Injection and ion source temperatures were set to 250°C and 300°C, respectively, with helium as the carrier gas at a flow rate of 1.0 mL/min. The column temperature was maintained at 60°C for 5 minutes, then increased to 250°C and held there for 15 minutes. Ionization was set at 70 eV with a 0.7 kV ionization current. The essential oil components were identified based on MS response, retention time, peak area, and relative peak percentage, with mass spectra matched to the Wiley 7 library. Retention indices of n-alkanes (C9-C40) were also compared with NIST database entries and relevant literature (de Alencar Filho et al., 2017). Component percentages were averaged from GC and GC-MS peak areas, with data processed using the Thermo Scientific™ Dionex™ Chromeleon™ 7 software, version 7.3.

### 2.5 Microbial strains and growth conditions

The antimicrobial activity of CMEOs was evaluated against a range of clinical bacterial pathogens, including *Escherichia coli* (ATCC 25922), *Salmonella typhi* (MTCC 3216), and *Staphylococcus aureus* (ATCC 25923). Post-harvested fungal pathogens isolated from decayed fruits included *Alternaria alstroemeriae* (BG3; PP594937, Source: Black grapes), *Fusarium fujikuroi* (BN1; PP594939, Source: Banana), and *Colletotrichum queenslandicum* (PY1; PP594922, Source: Papaya). Bacterial and fungal cultures were maintained at 4°C and sub-cultured monthly to ensure viability. Prior to exposure to the essential oils, bacterial strains were incubated on Mueller-Hinton agar (MHA) at 37°C for 12–18 hours, while fungal strains were cultivated on Potato Dextrose Agar (PDA) at 26°C.

### 2.6 Antibacterial activity

#### 2.6.1 Screening with disc diffusion assay

The antimicrobial efficacy of CMEOs was evaluated using a modified disc diffusion method, adapted from previously documented protocols (Ambrosio et al., 2019). Essential oil solutions were prepared at a 90% (v/v) concentration, with acetone used as an emulsifying agent. For comparison, ampicillin (20 µg/disc) and streptomycin (20 µg/disc) were included as positive controls for Gram-positive (*Staphylococcus aureus*) and Gram-negative (*Escherichia coli* and *Salmonella typhi*) bacteria, respectively. Bacterial suspensions were prepared by diluting colonies in sterile saline (0.85%), adjusting the optical density to 0.08–0.1 at 625 nm to match the 0.5 McFarland standard, which approximates a cell concentration of 1–2 × 10^8^ CFU/mL. This suspension was then used to inoculate MHA plates. EO solutions, along with positive and negative controls (acetone), were added to sterile 6-mm paper discs (10 µL/disc, Whatman No. 3), which were then placed on the agar. After a 24-hour incubation at 37°C, zones of inhibition (ZI) around each disc were measured to assess antibacterial activity. Each test was conducted in triplicate to ensure accuracy.

#### 2.6.2 Evaluation of minimum inhibitory concentration and minimum bactericidal concentration

The MIC and MBC of CMEOs was assessed using a modified macro-broth dilution method as described by Ahn and Jun (2007) and Sakkas et al. (2016). Inocula were prepared from overnight bacterial cultures, adjusting the turbidity of the bacterial suspension to match the 0.5 McFarland standard in sterile saline, resulting in a concentration of approximately 1.5 × 10^8^ CFU/mL (Eliason, 1940). An initial EO stock solution (2 mg/mL) was prepared by dispersing essential oil in Mueller-Hinton broth (MHB) and thoroughly vortexing at room temperature. Serial dilutions of the EO stock were then made in Mueller-Hinton broth with 0.5% Tween 20, resulting in final EO concentrations of 2, 1, 0.5, 0.25, 0.125, and 0.05 mg/mL. A 10 µL portion of the bacterial suspension was added to each dilution, yielding a final bacterial concentration of 5 × 10^5^ CFU/mL in each tube. Following incubation at 37°C for 36–48 hours, the lowest concentration at which no visible bacterial growth occurred was noted as the MIC. For MBC determination, 10 µL samples from tubes showing no visible growth were plated on Mueller-Hinton agar and incubated at 37°C for an additional 24–48 hours. The MIC was recorded as the lowest EO concentration that reduced bacterial growth by 90%, while the MBC was defined as the lowest concentration that either showed a 99.9% reduction in bacterial viability or displayed no visible bacterial growth on the agar plates (Eliason, 1940).

### 2.7 Antifungal activity

#### 2.7.1 Screening with direct contact assay

The antifungal activity of CMEOs at various growth stages was assessed through a direct contact assay, following the protocol by Yexiao et al. (2020). A stock solution of EOs was prepared with 0.5% acetone to achieve final concentrations ranging from 0.25 to 2 mg/mL. Sterilized petri dishes (100 × 15 mm) were filled with PDA heated to 45°C, to which EO stock solutions were added. Negative controls were prepared using 0.5% acetone without EOs, while positive controls included 0.25 mg/mL of the fungicide Bavistin. A 6-mm mycelial plug from the active edge of a three-day-old fungal culture was positioned in the center of each petri dish. Plates were sealed with parafilm and incubated in the dark at 26 ± 2°C for 4–7 days. Each EO concentration was tested in triplicate to ensure accuracy. Mycelial growth inhibition (%) was calculated using the Equation (2).


(2) $$\text{Mycelial Growth Inhibition }\left(\%\right)=\left(1-\frac{\text{Radial Growth of Treatment }\left(\text{mm}\right)}{\text{Radial Growth of Control }\left(\text{mm}\right)}\right)\times 100$$

#### 2.7.2 Evaluation of minimum inhibitory concentration and minimum fungicidal concentration

The fungistatic and fungicidal activities of CMEOs was evaluated using the macro-dilution method as described by Balouiri et al. (2016). Serial dilutions of the oils were prepared in 0.5% acetone, with concentrations ranging from 0.25 to 2 mg/mL, in sterile tubes containing 5 mL of Potato Dextrose Broth (PDB). A spore suspension was added to each tube, with a final concentration of 0.4–5 × 10^5^ CFU/mL. Positive controls (Bavistin at 0.1–0.5 mg/mL) and negative controls (0.5% acetone) were included. Tubes were incubated at 26±2°C in darkness for 4–7 days, depending on the fungal species. The MIC was defined as the lowest concentration with no visible fungal growth following incubation. For the MFC, 100 μL of broth from tubes with no visible growth was plated onto fresh PDA and incubated for an additional 24–48 hours at 26 ± 2°C. The MFC was identified as the lowest concentration that eliminated ≥99.9% of the initial fungal population, indicated by no fungal growth on the plates. All tests were carried out in triplicate to ensure precision and reliability (Enayatifard et al., 2021).

### 2.8 Antioxidant activity

#### 2.8.1 DPPH free-radical scavenging assay

The antioxidant potential of CMEO was evaluated through the DPPH (2,2-diphenyl-1-picrylhydrazyl) assay, following the method outlined by Braca et al. (2001). To perform this, varying concentrations of EOs (0–50 mg/mL) and ascorbic acid (used as a standard reference) were mixed with a 0.004% DPPH solution. The mixtures were incubated for 30 minutes at 25°C, after which their absorbance was measured at 517 nm using a UV-Vis spectrophotometer. Ascorbic acid at concentrations of 0–30 µg/mL was used as the positive control, while the 0.004% DPPH solution in methanol served as the control, and methanol was used as the blank. The percentage of radical scavenging activity was determined using the Equation (3).


(3) $$\text{Radical Scavenging Activity }\left(\%\right)=\left(\frac{{\text{A}}_{\text{Control}}-{\text{A}}_{\text{Sample}}}{{\text{A }}_{\text{Control}}}\right)\times \;100$$

In this formula, A_control_ represents the absorbance of the DPPH solution without any sample, while A_sample_ corresponds to the absorbance of the sample mixed with the DPPH solution. The antioxidant capacity of the essential oil samples was further expressed as the IC_50_ value, which indicates the concentration of the EO necessary to achieve a 50% decrease in absorbance relative to the control.

#### 2.8.2 ABTS free-radical scavenging assay

The antioxidant activity of CMEO against ABTS radicals was assessed using the method described by Insawang et al. (2019). The ABTS radical cation was generated by combining a 7 mM ABTS solution with 2.45 mM potassium persulfate and allowing the mixture to react in the dark at room temperature for 6–8 hours. The EOs were dissolved in absolute methanol to prepare concentrations ranging from 0 to 50 mg/mL. For the assay, 50 µL of either the EO sample or Trolox (used as a standard) was combined with 1950 µL of the ABTS reagent, mixed thoroughly, and incubated in the dark at room temperature for 30 minutes. Absorbance readings were taken at 734 nm using a Shimadzu UV-1900i spectrophotometer, with methanol serving as the blank. The percentage scavenging activity was calculated using the previously mentioned formula (3), and the antioxidant capacity was expressed as IC_50_ values for both the CMEOs and Trolox. Each test was conducted in triplicate to ensure result reliability.

#### 2.8.3 β-Carotene/linoleic acid bleaching assay

The β-carotene bleaching assay for assessing the antioxidant activity of CMEO was conducted following the procedure by Wettasinghe and Shahidi (1999). To begin, 2 mL of a β-carotene solution (0.2 mg/mL in chloroform) was added to a round-bottom flask along with 20 µL of linoleic acid and 200 µL of Tween 20. The mixture was then evaporated at 40°C for 10 minutes to remove the chloroform, and the residue was dissolved in 100 mL of distilled water to create an emulsion. The EOs were diluted in methanol to obtain final concentrations ranging from 0 to 50 mg/mL. For the assay, 150 µL of each EO dilution was added to 1.5 mL of the prepared emulsion in test tubes. The absorbance at 470 nm was recorded against an emulsion without β-carotene as the blank. The test tubes were incubated at 50°C, and absorbance readings at 470 nm were taken at intervals over a 60-minute period using a spectrophotometer. The same procedure was followed for the positive control, ascorbic acid, in concentrations of 10–100 µg/mL. The percentage inhibition of β-carotene bleaching was calculated using the Equation (4).


(4) $$\%\;\text{β}-\text{Carotene Bleaching Inhibition}=\left(\frac{\text{Initial Absorbance}-\text{Sample Absorbance}}{\text{Initial Absorbance}-\text{Control Absorbance}}\right)\times 100$$

In formula, initial absorbance (abs) is the absorbance of freshly preparing the emulsion at 0 minutes (t=0 min) min at 470 nm. The control absorbance is abs of emulsion without β-carotene. The sample absorbance is absorbance measured after 60 minutes (t=60 min) of adding EO or standard antioxidant.

The IC_50_ value (the concentration of EO that provides 50% inhibition) is calculated by plotting the inhibition percentages against different concentrations of the essential oils. The IC_50_ represents the concentration at which the antioxidants exhibit 50% reduction of oxidation in the system.

### 2.9 Statistical analysis

OriginPro software was utilized for statistical analysis. Descriptive statistics, including the calculation of means (n=3) and standard deviations (SD), were reported as mean ± SD for each treatment group. To evaluate statistical significance among groups, both one-way and two-way ANOVA were applied. One-way ANOVA was used to compare the means among the different essential oil treatments, while two-way ANOVA examined interaction effects between growth stages and variations in bioactivity. Following ANOVA, the Tukey test was performed to pinpoint significant differences between group means. Principal Component Analysis (PCA) was conducted to demonstrate how various growth stages of the plant influenced the bioactivity of the essential oils. All experimental procedures were conducted in triplicate to enhance reliability and reproducibility. The normal distribution of the data was checked before analysis, and statistical significance was defined at a p-value of less than 0.05 for all tests. IC_50_ values were determined using non-linear regression analysis with a sigmoidal dose-response model.

## 3 Result

### 3.1 Volatile oil yield and GC-MS analysis

**Figure 1:**
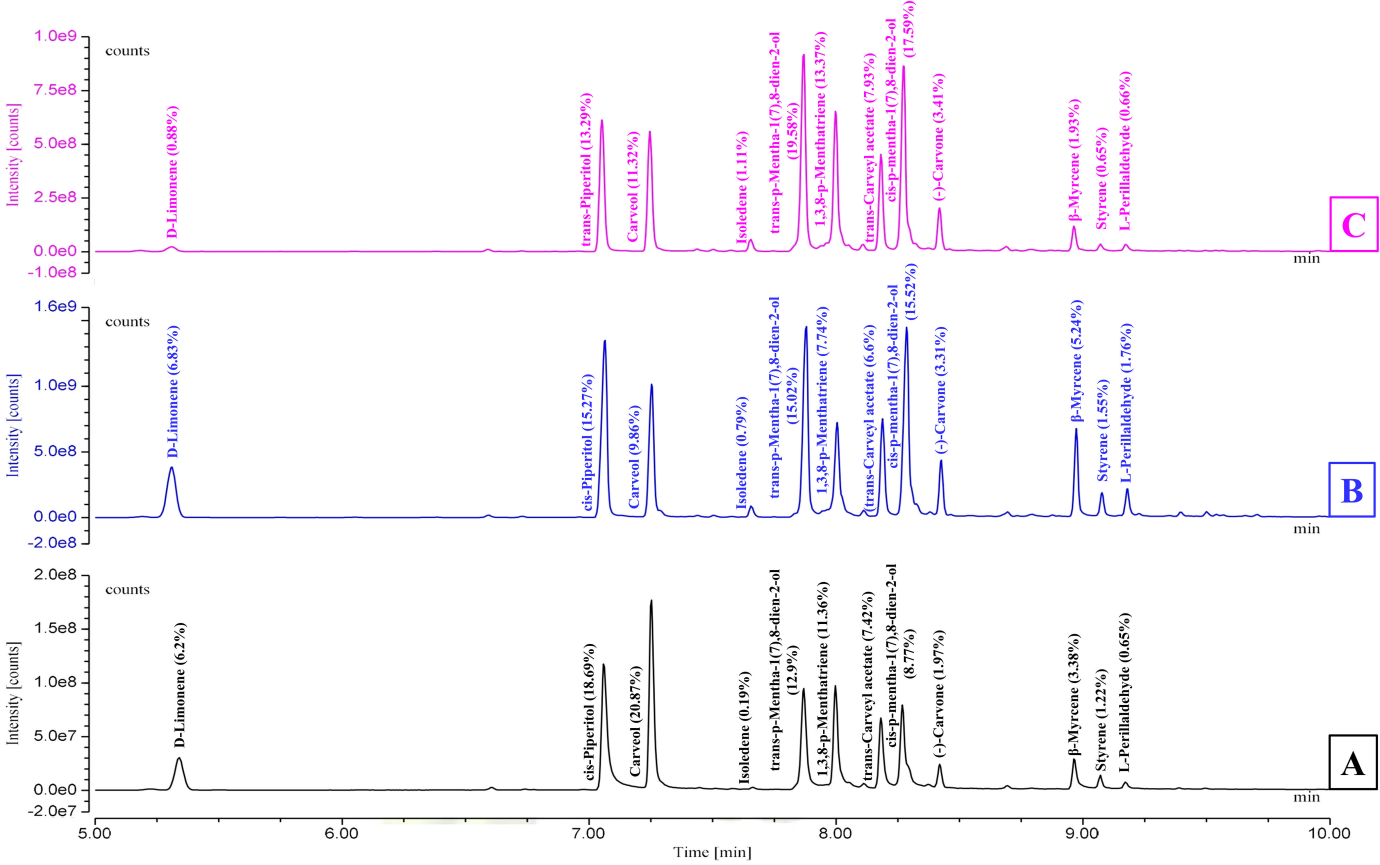
GC-MS chromatograms illustrating the chemical profile of *Cymbopogon martinii* essential oils across growth stages. **(A)** Vegetative stage, **(B)** Reproductive stage, and **(C)** Post-Reproductive stage. Chromatograms highlight the variation in compound composition as the plant matures.

As shown in **Table 1** and **Figure 1**, the essential oils (EOs) isolated from *C. martinii* at different growth stages, vegetative, reproductive, and post-reproductive, exhibited significant variations in yield, composition, and appearance. CMEOs color transitioned from olive (vegetative) to pale yellow (reproductive) and dark (post-reproductive) as seen in **Supplementary Figure S1**. Oil yield was highest during the reproductive stage (0.73%), compared to the vegetative (0.35%) and post-reproductive (0.53%) stages. GC-MS analysis identified a total of 59 compounds across all stages. The reproductive phase had the highest number of constituents (49; 97.86%), followed by the post-reproductive (42; 98.70%) and vegetative (30; 99.77%) stages. In the vegetative stage, major compounds included carveol (20.87%), trans-p-mentha-1(7),8-dien-2-ol (12.9%), cis-p-mentha-1(7),8-dien-2-ol (8.77%), trans-carveyl acetate (7.42%), D-limonene (6.2%), and β-myrcene (3.38%). During the reproductive stage, the dominant constituents shifted to cis-piperitol (15.27%), cis-p-mentha-1(7),8-dien-2-ol (15.52%), carveol (9.86%), D-limonene (6.83%), trans-carveyl acetate (6.6%), and carvone (3.31%). In the post-reproductive stage, trans-p-mentha-1(7),8-dien-2-ol (19.58%), cis-p-mentha-1(7),8-dien-2-ol (17.59%), cis-piperitol (13.29%), carveol (11.32%), trans-carveyl acetate (7.93%), and carvone (3.41%) predominated. Across all stages, oxygenated monoterpenes constituted the majority (75–80%), followed by monoterpene hydrocarbons (15–20%), and a minor fraction (1–2%) of non-terpenoid compounds, including aromatic compounds, ethers, esters, alkane derivatives, and chalcones. Stage-specific variation in compound abundance and distribution was effectively visualized using a heat map (**Supplementary Figure S2**), clearly illustrating the dynamic changes in EO composition during plant development.

**Table 1:** The chemical profile of the essential oils of *Cymbopogon martinii* at different growth stages.

S. No.	Name of Compounds	CAS NO.	Molecular Weight	Molecular Formula	RI^a^	RI^b^	Vegetative		Reproductive		Post-Reproductive	
							RT	Area (%)	RT	Area (%)	RT	Area (%)
1	Styrene	100-42-5	104.15	C_8_H_8_	898	897	9.07	1.22 ± 0.11	9.08	1.55 ± 0.16	9.07	0.65 ± 0.05
2	Camphene	78-81-4	136.23	C_10_H_16_	928	931	3.11	0.31 ± 0.14	3.08	0.21 ± 0.10	-	-
3	trans-Decalin	87-46-4	148.25	C_10_H_18_	973	972	8.06	0.74 ± 0.09	8.06	0.24 ± 0.03	8.05	0.55 ± 0.04
4	β-Myrcene	123-35-3	136.22	C_10_H_16_	990	992	8.96	3.38 ± 0.37	8.97	5.24 ± 0.35	8.96	1.93 ± 0.29
5	p-Cymene	99-87-7	134.22	C_9_H_12_	1015	1017	6.61	0.28 ± 0.05	6.59	0.20 ± 0.11	6.59	0.24 ± 0.15
6	o-Cymene	95-63-6	134.22	C_9_H_12_	1021	1023	5.22	0.26 ± 0.05	5.19	0.17 ± 0.13	-	-
7	D-Limonene	138-86-3	136.24	C_10_H_16_	1024	1025	5.34	6.20 ± 0.35	5.31	6.83 ± 0.55	5.31	0.88 ± 0.13
8	1,3,8-p-Menthatriene	634-67-4	136.22	C_10_H_14_	1078	1079	8	11.36 ± 1.55	8	7.74 ± 1.08	8	13.37 ± 1.43
9	cis-Linalool oxide	120-81-0	152.25	C_10_H_16_O	1102	1103	-	-	9.7	0.18 ± 0.12	-	-
10	Limonene oxide	75-64-7	136.22	C_10_H_16_O	1123	1123	7.45	0.08 ± 0.10	7.44	0.19 ± 0.05	7.44	0.28 ± 0.10
11	2,6-Dimethyl-1,3,5,7-octatetraene, E,E-	537-70-6	152.24	C_10_H_14_	1130	1128	-	-	7.13	0.34 ± 0.10	-	-
12	Cosmene	20242-35-9	204.36	C_15_ H_24_	1130	1129	-	-	-	-	5.18	0.23 ± 0.04
13	cis-p-Mentha-2,8-dien-1-ol	13224-60-6	152.25	C_10_H_16_O	1138	1136	-	-	8.54	0.12 ± 0.07	8.55	0.10 ± 0.13
14	4-Pentenylbenzene	25363-82-4	114.19	C_10_H_12_	1140	1141	-	-	11.22	0.08 ± 0.05	-	-
15	Isopinocarveol	139-10-3	152.25	C_10_H_14_O	1150	1149	-	-	8.46	0.15 ± 0.12	8.46	0.15 ± 0.06
16	β-Cyclocitral	93-83-6	136.21	C_10_H_14_O	1152	1153	-	-	14.44	0.25 ± 0.15	14.44	0.16 ± 0.15
17	Ethanone, 1-(3-methylphenyl)-	123-91-1	136.21	C_9_H_10_O	1157	1156	-	-	7.83	0.20 ± 0.06	-	-
18	4-Isopropenylcyclohexanone	587-46-7	152.25	C_10_H_14_O	1161	1163	-	-	7.5	0.19 ± 0.04	-	-
19	trans-Verbenol	15596-27-9	152.25	C_10_H_16_O	1166	1169	6.98	0.05 ± 0.11	6.96	0.06 ± 0.10	6.96	0.07 ± 0.10
20	Myrtenal	86-02-8	136.22	C_10_H_14_O	1170	1172	6.74	0.07 ± 0.03	6.73	0.10 ± 0.12	-	-
21	trans-p-mentha-1(7),8-dien-2-ol	20822-58-8	136.22	C_10_H_16_O	1185	1187	7.87	12.90 ± 1.90	7.88	15.02 ± 1.17	7.87	19.58 ± 1.84
22	Myrtenol	86-02-8	136.22	C_10_H_14_O	1192	1194	-	-	8.69	0.32 ± 0.11	8.69	0.47 ± 0.04
23	cis-Piperitol	3729-82-3	152.25	C_10_H_16_O	1197	1199	-	-	7.06	18.69 ± 1.90	7.06	15.27 ± 1.43
24	Pulegone	89-82-8	152.25	C_10_H_16_O	1201	1202	-	-	-	-	7.5	0.23 ± 0.13
25	Carveol	97-63-3	152.25	C_10_H_16_O	1203	1204	7.25	20.87 ± 2.78	7.25	9.86 ± 0.54	7.25	11.32 ± 1.03
26	1,6-Dihydrocarveol	10428-17-5	152.25	C_10_H_16_O	1204	1203	-	-	7.94	0.40 ± 0.05	7.94	0.47 ± 0.12
27	trans-Piperitol	3729-83-4	152.25	C_10_H_16_O	1205	1207	-	-	-	-	7.05	13.29 ± 0.95
28	trans-Isopiperitenol	13248-42-5	152.25	C_10_H_16_O	1210	1212	-	-	7.29	0.57 ± 0.04	7.29	0.32 ± 0.13
29	cis-Verbenone	88-86-5	152.25	C_10_H_14_O	1218	1218	-	-	8.88	0.10 ± 0.15	8.87	0.08 ± 0.10
30	cis-Dihydrocarvone	15058-73-5	152.25	C_10_H_14_O	1220	1223	7.96	0.65 ± 0.05	7.96	0.36 ± 0.09	7.96	0.69 ± 0.05
31	3-Cyclohexene-1-acetaldehyde, α,4-dimethyl-	642-73-0	136.21	C_10_H_14_O	1222	1223	-	-	8.11	0.37 ± 0.10	-	-
32	Cyclohexene, 4-isopropenyl-1-methoxymethoxymethyl-	51115-67-4	196.29	C_12_H_20_O_2_	1227	1228	8.79	0.15 ± 0.05	8.79	0.27 ± 0.12	8.79	0.25 ± 0.08
33	2-Cyclohexen-1-ol, 2-methyl-5-(1-methylethenyl)-, cis-	762-80-5	154.25	C_10_H_16_O	1229	1230	-	-	9.4	0.40 ± 0.10	-	-
34	cis-p-Mentha-1(7),8-dien-2-ol	14055-33-0	202.31	C_14_H_22_O_2_	1231	1232	8.27	8.77 ± 0.60	8.29	15.52 ± 2.19	8.27	17.59 ± 2.21
35	Carvomenthenal	1195-33-8	152.25	C_10_H_14_O	1232	1233	-	-	-	-	8.11	0.51 ± 0.05
36	Piperitone	88-73-1	152.25	C_10_H_16_O	1233	1235	9.39	0.16 ± 0.07	-	-	9.39	0.05 ± 0.06
37	L-Perillaldehyde	495-75-8	136.22	C_10_H_16_O	1237	1236	9.17	0.65 ± 0.07	9.18	1.76 ± 0.23	9.17	0.66 ± 0.09
38	trans-Carveyl acetate	67435-59-4	176.27	C_13_H_20_	1238	1239	8.18	7.42 ± 0.69	8.19	6.60 ± 0.60	8.18	7.93 ± 0.67
39	Carvone	60-33-3	218.34	C_18_H_32_O_2_	1243	1245	8.42	1.97 ± 0.16	8.43	3.31 ± 0.28	8.42	3.41 ± 0.48
40	5,7-Dodecadiyn-1,12-diol	21341-31-4	184.28	C_12_H_16_O_2_	1282	1284	-	-	9.66	0.11 ± 0.14	-	-
41	(S)-Perillyl alcohol	1408-95-3	136.22	C_10_H_16_O	1297	1299	8.9	0.06 ± 0.10	8.9	0.06 ± 0.07	-	-
42	Oxycymol	499-75-2	150.22	C_10_H_14_O	1298	1297	-	-	-	-	9	0.16 ± 0.13
43	α-Methylcinnamaldehyde	118-73-1	136.21	C_10_H_10_O	1330	1332	8.69	0.31 ± 0.05	-	-	-	-
44	Bicyclo[3.1.1]hept-3-ene-spiro-2,4’-(1’,3’-dioxane), 7,7-dimethyl-	101-40-6	194.27	C_12_H_18_O_2_	1351	1350	-	-	9.54	0.16 ± 0.13	-	-
45	Eugenol	102-99-4	136.22	C_10_H_16_O	1362	1364	8.3	1.95 ± 0.15	8.33	0.90 ± 0.12	8.32	0.76 ± 0.07
46	Isoledene	593-84-0	204.36	C_15_ H_24_	1377	1377	7.66	0.19 ± 0.07	7.66	0.79 ± 0.08	7.65	1.11 ± 0.07
47	Ageratriol	15401-90-2	228.35	C_15_ H_24_O	1513	1514	-	-	14.76	0.23 ± 0.14	14.75	0.16 ± 0.05
48	Isoelemicin	487-11-6	194.26	C_12_H_18_O_2_	1581	1585	7.58	0.08 ± 0.08	-	-	7.57	0.13 ± 0.03
49	Humulenol-II	1084-35-1	220.35	C_15_ H_24_O	1584	1585	-	-	-	-	8.73	0.06 ± 0.03
50	Methyl 3,5-tetradecadiynoate	2184-32-5	270.34	C_16_H_18_O_2_	1653	1652	-	-	-	-	9.54	0.06 ± 0.07
51	Bergamotol, Z-a-trans-	88034-74-6	220.35	C_15_ H_24_O	1693	1694	-	-	7.41	0.08 ± 0.04	7.4	0.08 ± 0.04
52	Methyl 7,9-octadecadiynoate	2184-36-9	296.42	C_20_H_26_O_2_	1837	1838	9.49	0.11 ± 0.12	-	-	-	-
53	Methyl 8,10-octadecadiynoate	2184-39-2	296.42	C_20_H_26_O_2_	1967	1969	9.22	0.05 ± 0.05	9.22	0.38 ± 0.04	9.22	0.12 ± 0.09
54	Methyl 4,6-tetradecadiynoate	2184-33-6	270.34	C_16_H_18_O_2_	1987	1988	-	-	9.57	0.17 ± 0.11	9.56	0.06 ± 0.08
55	2,5-Octadecadiynoic acid, methyl ester	20822-59-9	136.22	C_10_H_16_O	2078	2076	-	-	8.62	0.05 ± 0.14	8.61	0.05 ± 0.04
56	10,13-Octadecadiynoic acid, methyl ester	2184-43-5	296.42	C_20_H_26_O_2_	2085	2086	-	-	14.01	0.06 ± 0.05	-	-
57	Methyl linoleate	97-53-0	164.2	C_10_H_12_O_2_	2086	2087	8.38	0.40 ± 0.09	8.38	0.36 ± 0.06	8.38	0.29 ± 0.09
58	Eicosapentaenoic acid (EPA)	104963-17-2	302.45	C_20_H_30_O_2_	2138	2139	-	-	9.5	0.36 ± 0.11	9.5	0.06 ± 0.11
59	6,9,12,15-Docosatetraenoic acid, methyl ester	108-55-5	342.52	C_22_H_34_O_2_	2234	2235	-	-	15.76	0.05 ± 0.05	-	-
Total identified (%)							99.77%		97.86%		98.70%	
Yield (mL/kg)							3.5 ± 0.4 (0.35%)		7.33 ± 0.40 (0.73%)		5.33 ± 0.25 (0.53%)	
Monoterpene hydrocarbons (%)							21.49		20.19		16.65	
Oxygenated monoterpenes (%)							75.16		75.88		78.74	
Sesquiterpene hydrocarbons (%)							1.01		1.37		0.68	
Oxygenated sesquiterpenes (%)							0		0.08		0.14	
Miscellaneous Compounds (%)							2.25		2.39		2.49	

^a^Literature Retention index (Adams, 2007); ^b^calculated retention indices (DB-5 column).

### 3.2 Antibacterial activity

**Figure 2:**
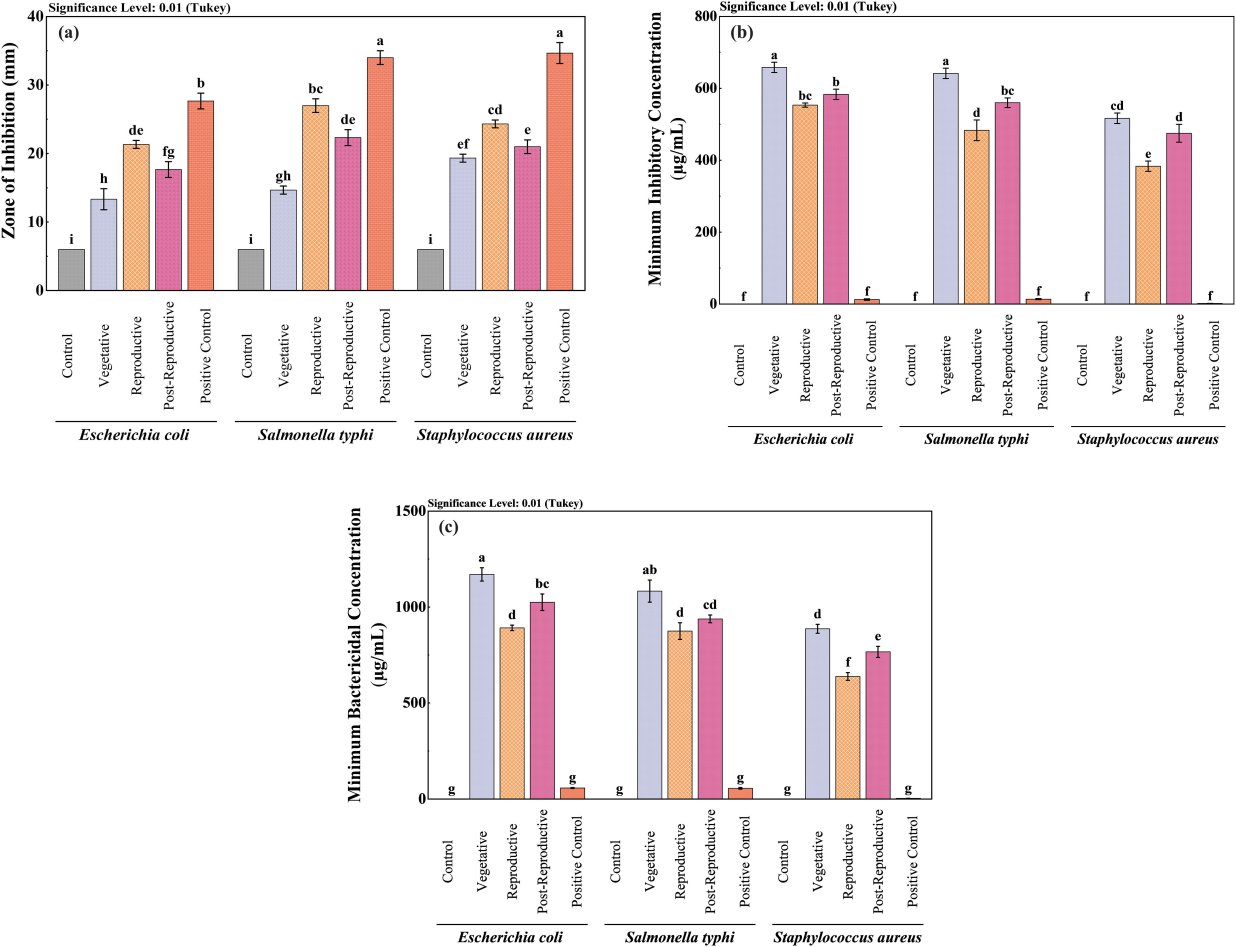
Comparative antibacterial efficacy of *Cymbopogon martinii* essential oils across growth stages. Bar graphs show **(a)** Zone of inhibition (ZI), **(b)** Minimum Inhibitory Concentration (MIC), and **(c)** Minimum Bactericidal Concentration (MBC). Positive control (ampicillin, streptomycin) was included for all assays to benchmark the efficacy of the essential oils. Significant differences across stages are denoted (p< 0.01), reflecting potency shifts in antibacterial properties at each growth stage. The lowercase letters indicate significant differences among the mean values as determined by Tukey’s test (p < 0.01). Means sharing the same letter are not significantly different, whereas means with different letters differ significantly.

The antibacterial activity of CMEOs from vegetative, reproductive, and post-reproductive stages was evaluated against *S. aureus*, *E. coli*, and *S. typhi*. Significant differences (P < 0.0001) were observed across growth stages and bacterial strains (**Supplementary Figure S3**), reflecting the variation in CMEOs chemical profiles. Reproductive-stage CMEO exhibited the highest antibacterial efficacy, with the largest ZI and lowest MIC and MBC values (**Table 2**). Post-reproductive CMEO showed moderate activity, while vegetative-stage CMEO was least effective. For *E. coli*, ZI was 27.7 mm in the reproductive stage, compared to 17.7 mm and 15.3 mm in post-reproductive and vegetative stages, respectively. *S. typhi* showed ZIs of 35.7 mm, 24.3 mm, and 19.3 mm across the respective stages. *S. aureus* followed the same trend: 33.7 mm (reproductive), 22.3 mm (post-reproductive), and 14.7 mm (vegetative) (**Figure 2a**). MIC and MBC values supported these findings (**Figures 2b, c**). *S. aureus* was the most sensitive strain across all stages, with an MIC of 383.3 µg/mL and MBC of 638.3 µg/mL during the reproductive stage. *S. typhi* and *E. coli* exhibited higher MICs (483.3 and 533.3 µg/mL) and MBCs (875.0 and 891.7 µg/mL, respectively). This pattern persisted in the other two growth stages, with *E. coli* consistently showing the lowest susceptibility. Although the positive control exhibited stronger antibacterial effects, CMEOs demonstrated considerable efficacy. Correlation analysis (**Supplementary Figure S4**) revealed a negative relationship between MIC values and key compounds such as trans-p-mentha-2,8-dien-1-ol, carvone, and D-limonene, suggesting their contribution to antibacterial activity.

**Table 2:** Antibacterial activity: minimum inhibitory concentration (MIC), minimum bactericidal concentration (MBC), and zone of inhibition for essential oils from different growth stages of *Cymbopogon martini*.

Bacterial Strain		Treatment Groups	Zone of Inhibition (mm)	MIC (µg/mL)	MBC (µg/mL)
Gram-negative	*Escherichia coli* (ATCC 25922)	Control	6.00 ± 0.00 ***^i^***	–	–
		Vegetative	13.33 ± 1.53 ***^h^***	658.3 ± 14.43 ***^f^***	1170.0 ± 34.64 ***^a^***
		Reproductive	21.33 ± 0.58 ***^de^***	533.3 ± 5.77 ***^bc^***	891.7 ± 14.43 ***^d^***
		Post-Reproductive	17.67 ± 1.15 ***^fg^***	583.3 ± 14.43 ***^b^***	1025.0 ± 43.30 ***^bc^***
		Positive Control (streptomycin)	27.67 ± 1.15 ***^b^***	13.66 ± 1.52 ***^f^***	57.33 ± 2.51g
	*Salmonella typhi* (MTCC 3216)	Control	6.00 ± 0.00 ***^i^***	–	–
		Vegetative	14.67 ± 0.58 ***^gh^***	641.7 ± 14.43 ***^a^***	1083.3 ± 57.74 ***^ab^***
		Reproductive	27.00 ± 1.00 ***^bc^***	483.3 ± 28.87 ***^d^***	875.0 ± 43.30 ***^d^***
		Post-Reproductive	22.33 ± 1.15 ***^de^***	560.0 ± 13.23 ***^bc^***	938.3 ± 20.21 ***^cd^***
		Positive Control (streptomycin)	34.00 ± 1.00 ***^a^***	12.66 ± 2.08 ***^f^***	55.66 ± 4.16g
Gram-Positive	*Staphylococcus aureus* (ATCC 25923)	Control	6.00 ± 0.00 ***^i^***	–	–
		Vegetative	19.33 ± 0.58 ***^ef^***	516.7 ± 14.43 ***^cd^***	886.7 ± 23.09 ***^d^***
		Reproductive	24.33 ± 0.58 ***^cd^***	383.3 ± 14.43 ***^e^***	638.3 ± 20.21*^f^*
		Post-Reproductive	21.00 ± 1.00 ***^e^***	475.0 ± 25 ***^d^***	766.7 ± 28.87 ***^e^***
		Positive Control (streptomycin)	34.67 ± 1.53 ***^a^***	1.66 ± 0.28 ***^f^***	3.5 ± 0.25 ***^g^***

Mean values ± SD (n=3) for MIC, MBC, and zone of inhibition, with significant differences (p<0.01).The superscript letters indicate significant differences among mean values as determined by Tukey’s test (p < 0.01). Values sharing the same letter are not significantly different, while those with different letters differ significantly.

### 3.3 Antifungal activity

**Figure 3:**
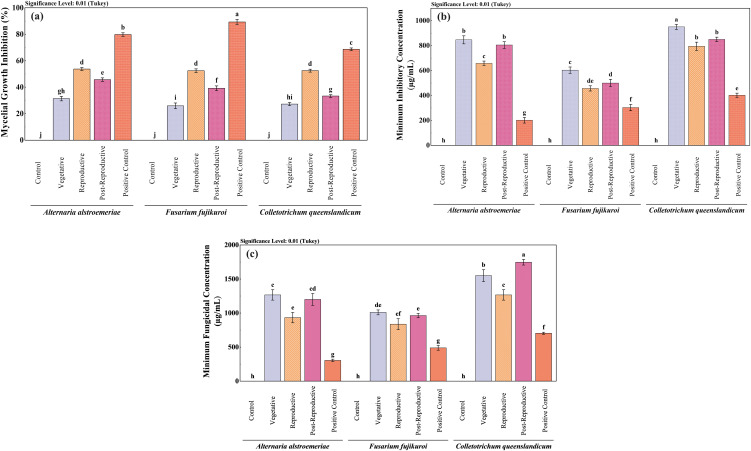
Comparative antifungal activity of *Cymbopogon martinii* essential oils from different growth stages. Bar graphs depict **(a)** Mycelial growth inhibition, **(b)** Minimum Inhibitory Concentration (MIC), and **(c)** Minimum Fungicidal Concentration (MFC). Positive control (Bavistin) was used as a reference standard for all assays. Significant differences (p< 0.01) illustrate variations in antifungal effectiveness of the oils as the plant matures.

The antifungal activity of CMEOs from vegetative, reproductive, and post-reproductive stages was evaluated against *C. queenslandicum*, *F. fujikuroi*, and *A. alstroemeriae* (**Supplementary Figure S5**). As shown in **Table 3** and **Figure 3**, variations in CMEO composition significantly influenced fungal growth inhibition, MIC, and MFC (P < 0.0001). Among the tested strains, *A. alstroemeriae* was the most sensitive. Inhibition increased from 31.36% (vegetative) to 53.70% (reproductive), before declining to 45.72% (post-reproductive). MIC values followed the same trend: 850 µg/mL (vegetative), 660 µg/mL (reproductive), and 810 µg/mL (post-reproductive). Corresponding MFC values were 1270, 930, and 1200 µg/mL, respectively. *F. fujikuroi* showed moderate sensitivity. The lowest inhibition (25.91%) and highest MIC (600 µg/mL) and MFC (1010 µg/mL) were recorded in the vegetative stage. The reproductive-stage CMEO exhibited the highest efficacy (52.50% inhibition, 500 µg/mL MIC, and 960 µg/mL MFC), while post-reproductive EO showed moderate improvements over the vegetative stage (39.21% inhibition, 500 µg/mL MIC, and 960 µg/mL MFC). *C. queenslandicum* was the least sensitive. Inhibition was 27.26% in the vegetative stage, peaking at 52.52% in the reproductive stage, then dropping to 33.32% in the post-reproductive stage. MIC and MFC values showed a similar pattern: highest in the vegetative stage (950 and 1270 µg/mL), lowest in the reproductive stage (800 and 1170 µg/mL), and intermediate in the post-reproductive stage (850 and 1250 µg/mL). Although the positive control showed substantially higher antifungal activity (**Figure 3**), CMEOs demonstrated meaningful inhibitory effects. Correlation analysis (**Supplementary Figure S4**) indicated strong negative correlations between MFC values and compounds such as (S)-perillyl alcohol, α-methylcinnamaldehyde, and isoeugenol, suggesting their potential contribution to antifungal activity against *A. alstroemeriae*, *F. fujikuroi*, and *C. queenslandicum*.

**Table 3:** Antifungal activity: percent growth inhibition, minimum inhibitory concentration (MIC), and minimum fungicidal concentration (MFC), of essential oils from different growth stages of *Cymbopogon martini*.

Fungal Stains	Treatment Groups	Growth Inhibition (%)	MIC (µg/mL)	MFC (µg/mL)
*Alternaria alstroemeriae* (BG3; PP594937)	Control	0.00 ± 0.00 ***^j^***	–	–
	Vegetative	31.36 ± 1.69 ***^gh^***	850.0 ± 32.0 ***^b^***	1270.0 ± 76.0 ***^c^***
	Reproductive	53.70 ± 1.14 ***^d^***	660.0 ± 18.0 ***^c^***	930.0 ± 76.0 ***^e^***
	Post-Reproductive	45.72 ± 1.33 ***^e^***	810.0 ± 26.0 ***^b^***	1200.0 ± 86.0 ***^cd^***
	Positive Control (Bavistin)	79.77 ± 1.33 ***^b^***	200.0 ± 23.0 ***^g^***	310.0 ± 18.0 ***^g^***
*Fusarium fujikuroi* (BN1; PP594939)	Control	0.00 ± 0.00 ***^j^***	–	–
	Vegetative	25.91 ± 2.17 ***^i^***	600.0 ± 25.0 ***^c^***	1010.0 ± 36.0 ***^de^***
	Reproductive	52.50 ± 1.39 ***^d^***	460.0 ± 21.0 ***^de^***	840.0 ± 81.0 ***^ef^***
	Post-Reproductive	39.21 ± 1.78 ***^f^***	500.0 ± 30.0 ***^d^***	960.0 ± 32.0 ***^e^***
	Positive Control (Bavistin)	89.27 ± 1.94 ***^a^***	310.0 ± 23.0 ***^f^***	490.0 ± 34.0 ***^g^***
*Colletotrichum queenslandicum* (PY1; PP594922)	Control	0.00 ± 0.00 ***^j^***	–	–
	Vegetative	27.26 ± 1.10 ***^hi^***	950.0 ± 20.0 ***^a^***	1550.0 ± 86.0 ***^b^***
	Reproductive	52.52 ± 1.12 ***^d^***	800.0 ± 33.0 ***^b^***	1270.0 ± 76.0 ***^c^***
	Post-Reproductive	33.32 ± 1.01 ***^g^***	850.0 ± 18.0 ***^b^***	1750.0 ± 42.0 ***^a^***
	Positive Control (Bavistin)	68.68 ± 1.00 ***^c^***	400.0 ± 18.0 ***^e^***	700.0 ± 15.0 ***^f^***

Mean values ± SD (n=3) for MIC, MFC, and percent growth inhibition, with significant differences (p<0.01).The superscript letters indicate significant differences among mean values as determined by Tukey’s test (p < 0.01). Values sharing the same letter are not significantly different, while those with different letters differ significantly.

### 3.4 Antioxidant activity

**Figure 4:**
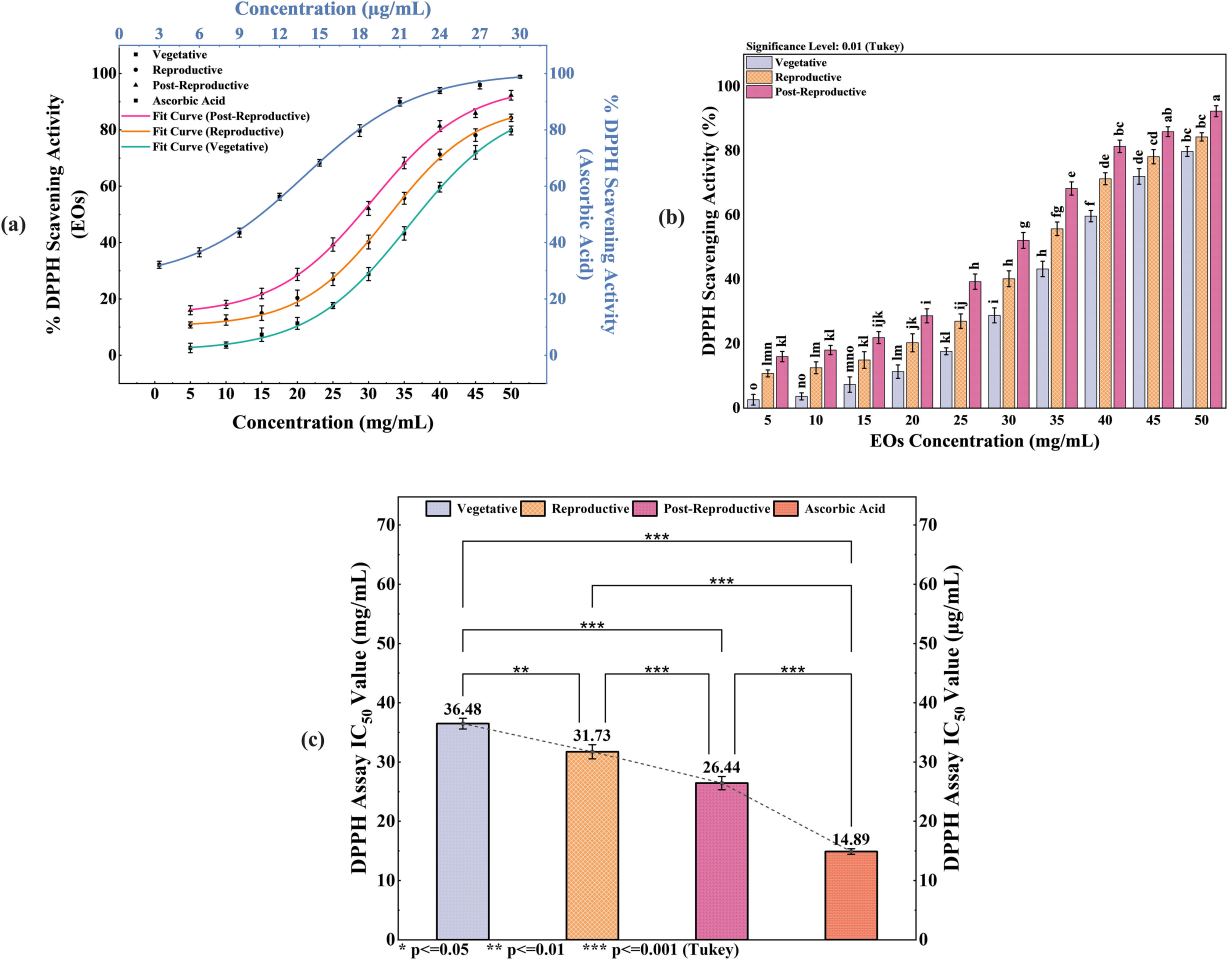
Antioxidant potential of *Cymbopogon martinii* essential oils at various growth stages via DPPH radical scavenging assay. **(a)** Dose–response curve showing % inhibition of DPPH radical by essential oils and ascorbic acid (Positive Control), used for IC_50_ calculation. **(b)** Statistical comparison of DPPH activity across growth stages (Tukey’s *post-hoc* test). **(c)** IC_50_ values indicating concentration for 50% inhibition, comparing essential oils to the standard. Bars marked with the same letter indicate no significant difference (p< 0.01). The symbols indicate levels of statisticalsignificance: ** = p < 0.01, *** = p < 0.001.

**Figure 5:**
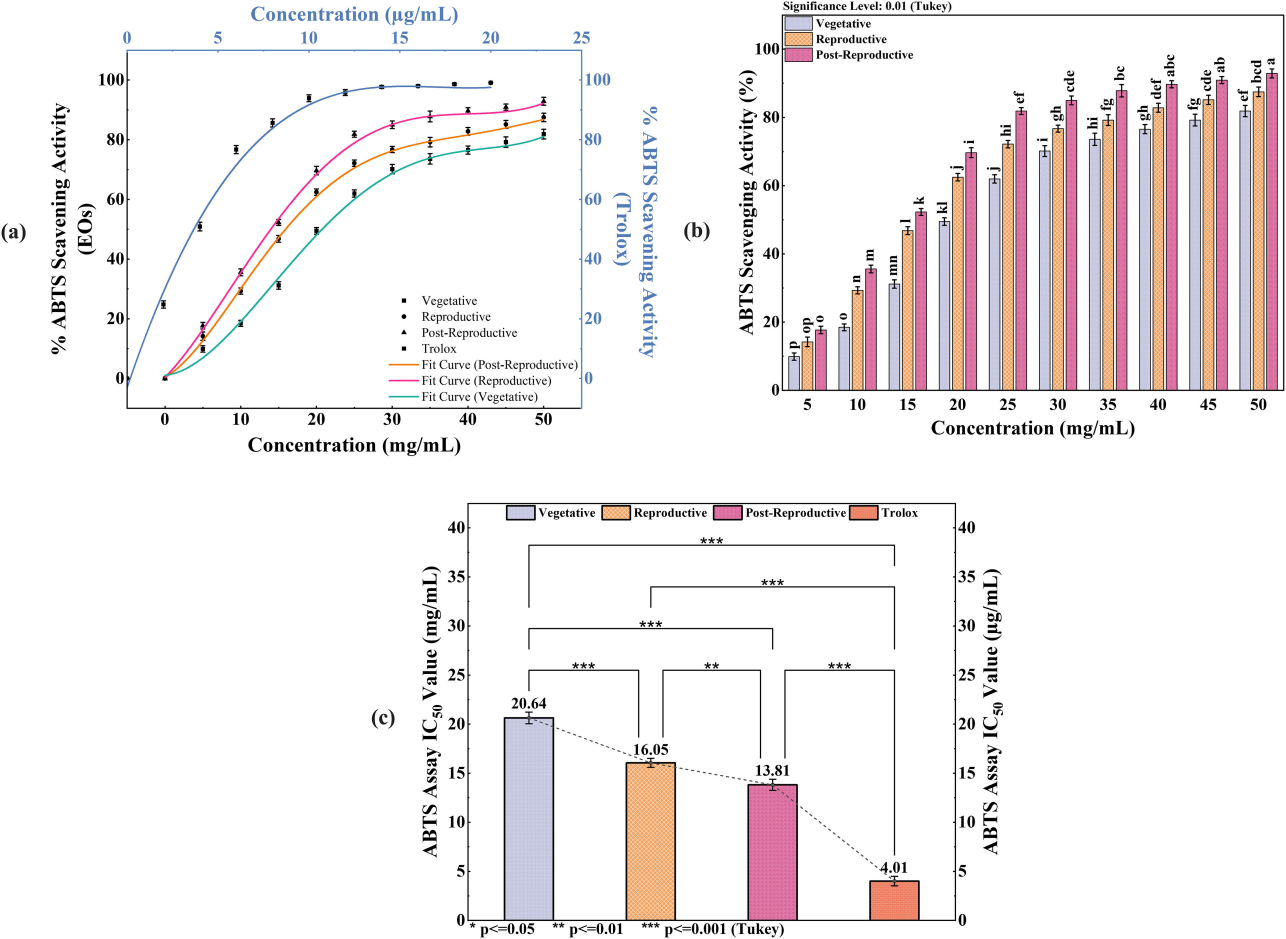
ABTS radical scavenging activity of *Cymbopogon martinii* essential oils across growth stages. **(a)** Dose–response curve showing % ABTS radical scavenging by essential oils and Trolox (Positive Control), used for IC_50_ calculation.**(b)** Comparative analysis of ABTS activity significance across growth stages (Tukey’s test). **(c)** IC_50_ values indicating the concentration required for 50% inhibition, comparing essential oils to Trolox. Bars with identical letters represent no significant difference (p< 0.01). The symbols indicate levels of statistical significance: ** = p < 0.01, *** = p < 0.001.

**Figure 6:**
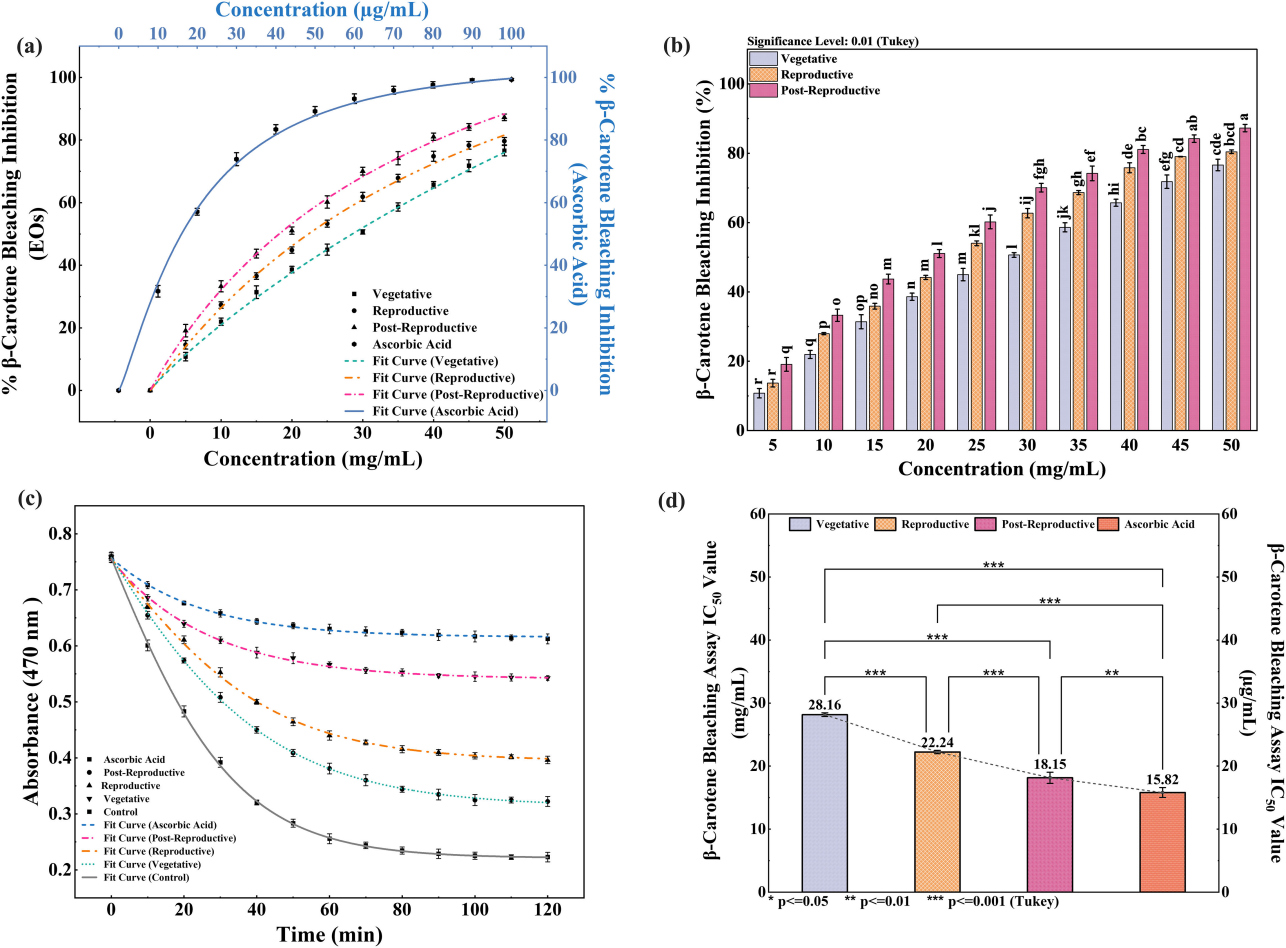
Inhibition of β-carotene degradation by *Cymbopogon martinii* essential oils at different growth stages. **(a)** Dose–response curve for % inhibition of β-carotene bleaching by essential oils and ascorbic acid (Positive Control), used for IC_50_ calculation. **(b)** Significance test across growth stages (Tukey’s analysis) for bleaching inhibition activity. **(c)** Rate of β-carotene degradation in the presence of essential oils compared to control. **(d)** IC_50_ values of essential oils and standard for bleaching inhibition. Identical letters on bars indicate no significant variation (p< 0.01). The symbols indicate levels of statistical significance: ** = p < 0.01, *** = p < 0.001.

The antioxidant activity of CMEOs from vegetative, reproductive, and post-reproductive stages was evaluated using DPPH, ABTS, and β-carotene bleaching assays, with significant variation observed across stages (P < 0.0001) (**Figures 4**–**6**). In the DPPH assay (**Figure 4**), post-reproductive EO exhibited the highest scavenging activity (92.29% at 50 mg/mL), followed by reproductive (84.29%) and vegetative (79.77%) stages. IC_50_ values followed the same trend: post-reproductive (26.44 mg/mL), reproductive (31.73 mg/mL), and vegetative (36.48 mg/mL). Ascorbic acid (standard) achieved 98.87% inhibition at 30 µg/mL with an IC_50_ of 14.89 µg/mL (**Table 4**). The ABTS assay (**Figure 5**) also showed stage-dependent activity. Post-reproductive EO demonstrated the strongest inhibition (92.90% at 50 mg/mL, IC_50_= 13.81 mg/mL), followed by reproductive (87.49%, IC_50_ = 16.05 mg/mL) and vegetative (81.87%, IC_50_ = 20.64 mg/mL). Trolox (standard) reached 92.90% inhibition at 20 µg/mL with an IC_50_ of 4.01 µg/mL (**Table 5**). In the β-carotene bleaching assay (**Figure 6**), inhibition ranged from 76.6% to 87.26%. The post-reproductive EO showed the highest activity (87.26%), followed by reproductive (79.64%) and vegetative (76.6%) stages. IC_50_ values were lowest in the post-reproductive EO (18.15 mg/mL), followed by reproductive (22.24 mg/mL) and vegetative (28.16 mg/mL) (**Table 6**). Ascorbic acid again showed strong activity (IC_50_ = 15.81 µg/mL). Kinetic monitoring of β-carotene degradation (**Figure 6c**) confirmed sustained inhibition over time, strongest in post-reproductive EO, followed by reproductive and vegetative stages. Overall, the antioxidant potential of CMEO increased progressively from vegetative to post-reproductive stages across all assays. Correlation analysis (**Supplementary Figure S4**) revealed strong negative correlations between IC_50_ values and compounds such as α-methylcinnamaldehyde, (S)-perillyl alcohol, and eugenol, indicating their key contribution to antioxidant activity.

**Table 4:** DPPH radical scavenging activity and IC_50_ of essential oils of *Cymbopogon martinii* at different growth stage and the standard.

Concentration (mg/mL)	% DPPH Scavenging Activity at Different Growth Stages			Concentration (µg/mL)	Positive Control (Ascorbic Acid)
	Vegetative	Reproductive	Post-Reproductive		
5	2.60 ± 1.66 ***°***	10.78 ± 1.07 ***^lmn^***	16.01 ± 1.60 ***^kl^***	3	32.11 ± 1.26
10	3.64 ± 1.11 ***^no^***	12.54 ± 1.84 ***^lm^***	18.04 ± 1.42 ***^kl^***	6	36.55 ± 1.59
15	7.32 ± 2.39 ***^mno^***	14.95 ± 2.59 ***^kl^***	21.91 ± 1.85 ***^ijk^***	9	43.51 ± 1.60
20	11.34 ± 2.10 ***^lm^***	20.32 ± 2.80 ***^jk^***	28.70 ± 2.17 ***^i^***	12	56.28 ± 1.25
25	17.66 ± 1.09 ***^kl^***	27.01 ± 2.25 ***^ij^***	39.29 ± 2.38 ***^h^***	15	68.33 ± 1.17
30	28.81 ± 2.32 ***^i^***	40.19 ± 2.47 ***^h^***	52.12 ± 2.45 ***^g^***	18	79.74 ± 2.07
35	43.26 ± 2.39 ***^h^***	55.73 ± 2.12 ***^fg^***	68.27 ± 2.05 ***^e^***	21	89.93 ± 1.45
40	59.67 ± 1.73 ***^f^***	71.29 ± 1.87 ***^de^***	81.37 ± 1.93 ***^bc^***	24	93.97 ± 1.05
45	72.02 ± 2.42 ***^de^***	78.14 ± 2.21 ***^cd^***	85.92 ± 1.53 ***^ab^***	27	95.97 ± 1.42
50	79.77 ± 1.54 ***^bc^***	84.29 ± 1.30 ***^bc^***	92.29 ± 1.71 ***^a^***	30	98.87 ± 0.45
**IC_50_**	**36.48 ± 0.90*^a^***	**31.73 ± 1.19*^b^***	**26.44 ± 1.12*^c^***	**IC_50_**	**14.89 ± 0.46*^d^***

Values represent means ± SD (n=3). The significant difference (p<0.01) is indicated by the letters (Tukey’s test).The bold values represent the IC₅₀ values, which indicate the concentration of essential oil (or standard antioxidant) required to achieve 50% inhibition.

**Table 5:** ABTS radical scavenging activity and IC_50_ of essential oils of *Cymbopogon martinii* at different growth stages and the standard.

Concentration (mg/mL)	% ABTS Scavenging Activity at Different Growth Stages			Concentration (µg/mL)	Positive Control (Trolox)
	Vegetative	Reproductive	Post-Reproductive		
5	9.89 ± 1.09 ***^p^***	14.19 ± 1.42 ***^op^***	17.69 ± 1.09 ***°***	2	24.81 ± 1.23
10	18.48 ± 1.00 ***°***	29.30 ± 1.03 ***^n^***	35.56 ± 1.14 ***^m^***	4	50.90 ± 1.46
15	31.16 ± 1.23 ***^mn^***	46.81 ± 1.15 ***^l^***	52.28 ± 1.00 ***^k^***	6	76.71 ± 1.36
20	49.46 ± 1.12 ***^kl^***	62.46 ± 1.08 ***^j^***	69.67 ± 1.43 ***^i^***	8	85.63 ± 1.35
25	61.98 ± 1.21 ***^j^***	72.16 ± 1.07 ***^hi^***	81.87 ± 1.00 ***^ef^***	10	93.91 ± 1.16
30	70.14 ± 1.58 ***^i^***	76.72 ± 1.03 ***^gh^***	85.00 ± 1.29 ***^cde^***	12	95.81 ± 0.99
35	73.59 ± 1.77 ***^hi^***	79.21 ± 1.58 ***^fg^***	87.81 ± 1.81 ***^bc^***	14	97.67 ± 0.44
40	76.56 ± 1.35 ***^gh^***	82.82 ± 1.30 ***^def^***	89.71 ± 1.03 ***^abc^***	16	97.99 ± 0.31
45	79.21 ± 1.73 ***^fg^***	85.16 ± 1.34 ***^cde^***	90.94 ± 1.04 ***^ab^***	18	98.62 ± 0.28
50	81.87 ± 1.65 ***^ef^***	87.49 ± 1.41 ***^bcd^***	92.90 ± 1.31 ***^a^***	20	99.10 ± 0.06
**IC_50_**	**20.64 ± 0.60*^a^***	**16.05 ± 0.46*^b^***	**13.81 ± 0.57*^c^***	**IC_50_**	**4.01 ± 0.48*^d^***

Values represent means ± SD (n=3). The significant difference (p<0.01) is indicated by the letters (Tukey’s test).The bold values represent the IC₅₀ values, which indicate the concentration of essential oil (or standard antioxidant) required to achieve 50% inhibition.

**Table 6:** β-Carotene bleaching inhibition activity and IC_50_ of essential oils of *Cymbopogon martinii* at different growth stage and the standard.

Concentration (mg/mL)	% β-Carotene Bleaching Inhibition at Different Growth Stages			Concentration (µg/mL)	Positive Control (Ascorbic Acid)
	Vegetative	Reproductive	Post-Reproductive		
5	10.78 ± 1.36 ***^r^***	14.57 ± 1.35 ***^r^***	19.08 ± 1.98 ***^q^***	10	31.7 ± 1.86
10	21.95 ± 1.17 ***^q^***	27.3 ± 1.03 ***^p^***	33.26 ± 1.77 ***°***	20	57.1 ± 1.09
15	31.39 ± 2.02 ***^op^***	36.55 ± 1.04 ***^no^***	43.72 ± 1.41 ***^m^***	30	73.88 ± 2.05
20	38.61 ± 1.04 ***^n^***	44.85 ± 1.01 ***^m^***	51.07 ± 1.16 ***^l^***	40	83.43 ± 1.52
25	44.99 ± 1.78 ***^m^***	53.29 ± 1.11 ***^kl^***	60.2 ± 1.98 ***^j^***	50	89.22 ± 1.49
30	50.64 ± 0.67 ***^l^***	61.86 ± 1.41 ***^ij^***	70.07 ± 1.24 ***^fgh^***	60	93.21 ± 1.54
35	58.62 ± 1.31 ***^jk^***	67.85 ± 1.21 ***^gh^***	74.19 ± 2.11 ***^ef^***	70	95.92 ± 1.24
40	65.72 ± 1.02 ***^hi^***	74.81 ± 1.60 ***^de^***	81.08 ± 1.15 ***^bc^***	80	97.64 ± 1.03
45	71.79 ± 1.94 ***^efg^***	78.3 ± 1.23 ***^cd^***	84.20 ± 1.09 ***^ab^***	90	99.0 ± 0.58
50	76.6 ± 1.67 ***^cde^***	79.64 ± 1.19 ***^bcd^***	87.26 ± 1.07 ***^a^***	100	99.34 ± 0.34
**IC_50_**	**28.16 ± 0.30*^a^***	**22.24 ± 0.26*^b^***	**18.15 ± 0.89*^c^***	**IC_50_**	**15.81 ± 0.79*^d^***

Values represent means ± SD (n=3). The significant difference (p<0.01) is indicated by the letters (Tukey’s test).The bold values represent the IC₅₀ values, which indicate the concentration of essential oil (or standard antioxidant) required to achieve 50% inhibition.

### 3.5 Principal component analysis

**Figure 7:**
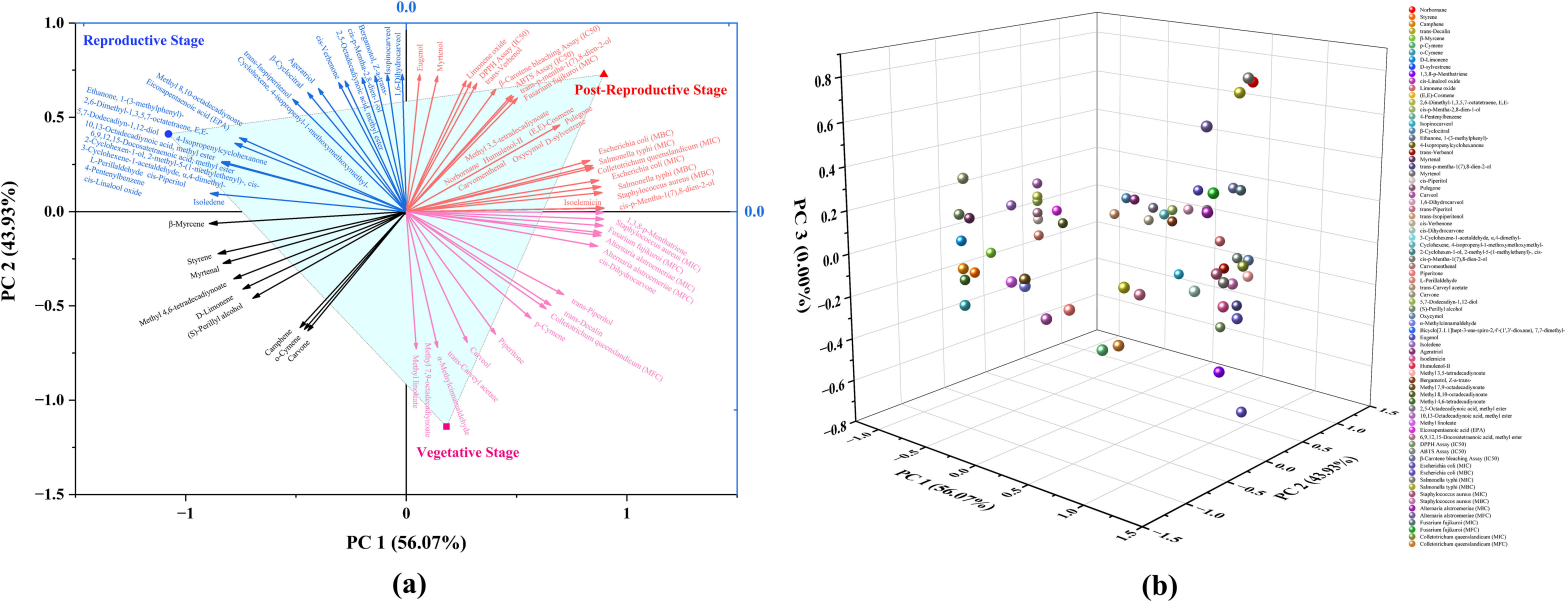
Principal Component Analysis (PCA) depicting the distribution and influence of chemical constituents and biological activities of essential oils from *Cymbopogon martinii* across three phenological stages. **(a)** Two-dimensional PCA biplot combining sample scores and variable loadings. Clear separation among vegetative, reproductive, and post-reproductive stages is evident, reflecting significant stage-specific differences in chemical composition and bioactivity. Principal Components 1 and 2 (PC1 and PC2) accounted for 56.07% and 43.93% of the total variance, respectively. **(b)** PCA loading plot illustrating the spatial distribution and contributions of individual compounds and biological activities to the principal components.

Principal component analysis (PCA) was performed to assess the chemical variation and bioactivity profile of CMEO across different growth stages. The analysis included the relative abundance (% area) of identified chemical constituents along with corresponding biological parameters (MIC, MBC, MFC, IC_50_). The PCA model demonstrated high statistical validity, accounting for 100% of the total variance. The first two principal components (PC1 and PC2) captured the full variability in the dataset, with PC1 explaining 56.07% and PC2 accounting for 43.93% of the total variance (**Supplementary Table S1**). The 2D biplot (**Figure 7a**) effectively visualizes the grouping of CMEO samples across different stages and their relationship with both chemical and biological variables. The loading plot (**Figure 7b**) further illustrates the contribution of individual variables to each principal component, supporting the interpretation of stage-specific chemical signatures and bioactivities. The associated eigenvalues were 42.61 for PC1 and 33.39 for PC2, confirming their strong explanatory power. Detailed loading values for individual compounds and activities are provided in **Supplementary Table S2**. Together, the PCA results underscore distinct compositional and bioactivity profiles of CMEO across growth stages, with clear separation and variable contributions effectively summarized by PC1 and PC2.

## 4 Discussion

### 4.1 Essential oil yield and compositional dynamics across growth stages

This study explored growth stage–dependent changes in the essential oil profile of wild-growing *C. martinii* and their relationship to antimicrobial and antioxidant activities. EO yield followed a bell-shaped trend, peaking during the reproductive stage, likely due to increased metabolic demand for volatiles involved in pollinator attraction, an observation consistent with other aromatic plants (Palá-Paúl et al., 2001). GC-MS analysis confirmed stage-specific quantitative variation in EO composition, particularly a broader activation of biosynthetic pathways during the reproductive phase. Such developmental modulation of EO output has been widely noted in aromatic species undergoing physiological transitions (Moghaddam and Mehdizadeh, 2017; Chrysargyris et al., 2021).

### 4.2 Shifts in major chemical classes: oxygenated *vs*. hydrocarbon monoterpenes

A developmental increase in oxygenated monoterpenes accompanied by a reduction in monoterpene hydrocarbons was observed, reflecting regulatory shifts in terpene metabolism. This pattern aligns with previous findings in *C. martinii* and other monoterpene-rich taxa (Rao et al., 2005; Verma et al., 2018), though past studies often overlooked stage-specific variation.

The transition from carveol dominance in the vegetative phase to cis-/trans-p-mentha-1(7),8-dien-2-ol in reproductive and post-reproductive stages suggests biochemical prioritization of compounds linked to reproductive signaling and defense, similar to trends reported in other species (Chu et al., 2013; Rathore et al., 2022).

### 4.3 Geographic, climatic influence, and chemotypic variation

Our results resemble the profile of *C. martinii* var. *sofia* reported from Rajasthan Jain and Sharma (2017), characterized by carveol-rich chemotypes rather than the geraniol-dominant var. *motia* (Raina et al., 2003; Rao et al., 2005). This chemotypic divergence likely reflects the influence of geography and wild-growing conditions on EO biosynthesis (Jangi et al., 2022).

### 4.4 Functional roles of key compounds

Growth stage–specific enrichment of compounds reveals their probable ecological functions. Carveol and cis-piperitol were more abundant in the vegetative phase, supporting roles in constitutive defense. Their decline over time coincided with increased levels of trans-/cis-p-mentha-1(7),8-dien-2-ol, likely linked to reproductive signaling or adaptive protection under stress. Stable levels of trans-carveyl acetate suggest a constitutive protective function across development. Carvone, which increased in the post-reproductive stage, is a well-established antimicrobial agent and may serve in late-stage defense enhancement (Bakrim et al., 2022).

### 4.5 Biosynthetic basis and environmental considerations

Key monoterpenes identified in *C. martinii* including carveol, carvone, D-limonene, and p-mentha-dien-2-ol isomers are synthesized via the plastidial MEP pathway from geranyl diphosphate (GPP), with downstream modifications by terpene synthases and cytochrome P450 monooxygenases (Dudareva et al., 2013; Vranová et al., 2013; Tholl, 2015). The increased abundance of oxygenated monoterpenes during later growth stages suggests developmentally regulated biosynthesis aligned with functions like reproductive signaling and oxidative stress mitigation. Although EO biosynthesis is influenced by environmental conditions such as rainfall and temperature, our sampling across identical phenological stages in two growing seasons minimized inter-annual variability. Averaged GC-MS data (mean ± SD) allowed a clearer focus on stage-specific metabolic trends. Future studies under controlled environments could further distinguish developmental effects from climatic factors.

### 4.6 Stage-dependent antibacterial activity

Antibacterial potency varied with growth stage, paralleling chemical composition. Reproductive-stage EOs showed the strongest activity, attributed to elevated cis- and trans-p-mentha-1(7),8-dien-2-ol, which destabilize bacterial membranes and inhibit enzymatic systems (Alitonou et al., 2012; Ambrosio et al., 2020, 2021). Post-reproductive oils, though still active, showed slightly reduced efficacy, likely due to decreased D-limonene, β-myrcene, and eugenol compounds known for disrupting bacterial respiration and membranes (Nuñez and D’Aquino, 2012; Han et al., 2020; Połeć et al., 2020; Gupta et al., 2021; Ulanowska and Olas, 2021). Their partial replacement by carvone and trans-carvyl acetate, which act through quorum sensing and enzyme inhibition (Pina et al., 2022), may have altered overall potency. Vegetative-stage oils exhibited the weakest antibacterial action, dominated by carveol and cis-piperitol, which act on microbial enzymes but may lack strong membrane-disruptive properties (Roman et al., 2023). Additionally, limited synergism among constituents may contribute to reduced efficacy (Kamatou et al., 2008; Tamgue et al., 2011). Susceptibility varied by strain: *S. aureus* was most sensitive, likely due to its permeable Gram-positive cell wall (Oussalah et al., 2007; Galgano et al., 2022), while Gram-negative *E. coli* and *S. typhi* were more resistant due to their outer membranes (Longbottom et al., 2004; Dai et al., 2013; Rashid et al., 2013). Slightly higher sensitivity of *S. typhi* may relate to differences in membrane structure or EO interactions (Azad, 2021).

### 4.7 Antifungal activity: stage-specific efficacy

The antifungal potential of CMEOs showed strong dependence on plant growth stage, primarily linked to the abundance of oxygenated monoterpenes. Vegetative-phase oils exhibited the weakest activity, despite carveol’s known ability to disrupt fungal membranes (Soković et al., 2006; Abbaszadeh et al., 2014). The limited efficacy may reflect insufficient synergy with other active compounds or inhibitory interactions (Boni, 2017; James and Ikani, 2023). In contrast, reproductive-stage oils demonstrated the strongest antifungal effects, coinciding with maximum concentrations of cis- and trans-p-mentha-1(7),8-dien-2-ol compounds that disrupt fungal membranes and induce cell leakage (Kostik and Bauer, 2015; Brahmi et al., 2021). Their interaction with ergosterol alters membrane fluidity and integrity (Busato de Feiria et al., 2016; Benomari et al., 2018; Soliman et al., 2022), and may also interfere with ergosterol biosynthesis (Mohamed Ibrahim et al., 2018; Scalese et al., 2024). Post-reproductive EOs retained moderate antifungal activity, supported by elevated levels of carveol, trans-carveyl acetate, and p-mentha-1(7),8-dien-2-ol isomers. These compounds likely act through multiple mechanisms, including membrane destabilization, glycolytic inhibition, and morphological disruption (Yu et al., 2022; Xiong et al., 2023). The reduced presence of D-limonene, β-myrcene, and cis-piperitol may have contributed to the slight decline in activity. Sensitivity varied by fungal strain. *A. alstroemeriae* was most susceptible, likely due to its more permeable membrane structure. *F. fujikuroi* showed intermediate response, while *C. queenslandicum* was least sensitive, possibly due to inherent resistance mechanisms like enhanced sterol biosynthesis and stress adaptation (Moutassem et al., 2019; Vasileva, 2023; Erdoğan, 2024).

### 4.8 Antioxidant potential and its chemical drivers

Antioxidant activity increased progressively from the vegetative to the post-reproductive stage, consistent across DPPH, ABTS, and β-carotene bleaching assays. Similar trends have been observed in other aromatic species such as *Foeniculum vulgare* and *Origanum vulgare*, where late-stage accumulation of active compounds enhances radical-scavenging potential (Salami et al., 2017; Morshedloo et al., 2018). Post-reproductive EO showed the highest antioxidant activity, attributed to elevated levels of cis- and trans-p-mentha-1(7),8-dien-2-ol, known for their radical stabilization and hydrogen-donating abilities (Sitzmann et al., 2014; Jordá Marín, 2018). Additional contributors included eugenol, D-limonene, 1,3,8-p-menthatriene, and carveol all recognized for their antioxidant efficacy (Niki, 2010; Gülçin, 2011; Mueller and Boehm, 2011; Serafim et al., 2021; Orlo et al., 2023). Trans-carveyl acetate may also enhance activity via hydrogen donation. In contrast, vegetative-stage EO showed the lowest antioxidant effect despite high carveol content, likely due to limited synergy with other active compounds. Reproductive-stage EO displayed moderate activity, supported by cis-piperitol and p-mentha-1(7),8-dien-2-ol, though lower levels of carvone and carveol may have reduced overall potency (Duarte-Almeida et al., 2006; Olszowy and Dawidowicz, 2018; Pombal et al., 2020). The antioxidant roles of β-myrcene and cis-piperitol remain underexplored and merit further study.

### 4.9 Multivariate analysis confirms stage-specific clustering

Principal component analysis (PCA) clearly differentiated EO samples by phenological stage, with vegetative, reproductive, and post-reproductive oils forming distinct clusters (**Figure 7a**). The loading plot (**Figure 7b**) revealed that antimicrobial compounds were strongly associated with reproductive-stage oils, while antioxidant-rich constituents clustered with post-reproductive samples. Vegetative EO appeared chemically intermediate.

The PCA model explained 100% of the variance (PC1 = 56.07%, PC2 = 43.93%) and was supported by eigenvalue and loading data (**Supplementary Tables S1, S2**), confirming its robustness. These results highlight the importance of the growth stage in determining both EO composition and bioactivity, offering a framework for selecting optimal harvest times based on intended applications of *C. martinii* essential oil.

## 5 Conclusion

The present study clearly demonstrates that the growth stage of wild-grown *Cymbopogon martinii* (Roxb.) Wats. significantly influences the essential oil’s chemical composition and bioactivity. A statistically significant shift (P < 0.0001) was observed from monoterpene hydrocarbons dominating the vegetative phase to an abundance of oxygenated monoterpenes in the reproductive and post-reproductive stages. These changes reflect adaptive physiological responses related to defense, reproductive signaling, and oxidative stress management. Correlation analysis identified key compounds such as carvone, D-limonene, (S)-perillyl alcohol, and α-methylcinnamaldehyde as strong contributors to enhanced antimicrobial, antifungal, and antioxidant activities. Biological assays confirmed that reproductive-stage oil was most effective against *Staphylococcus aureus* and *Alternaria alstroemeriae*, while post-reproductive oil exhibited the highest antioxidant activity. These findings underscore the importance of selecting the appropriate harvest stage to maximize essential oil quality for specific end uses. The results provide a practical framework for guiding the targeted use of *C. martinii* essential oil in pharmaceutical, cosmetic, and agricultural applications as a sustainable alternative to synthetic agents. Future work may explore cultivation practices and genotype selection to further optimize bioactive profiles for commercial exploitation.

## Data Availability

The original contributions presented in the study are included in the article/**Supplementary Material**. Further inquiries can be directed to the corresponding author.
