# Supplementary material for: Influence of growth stage on the chemical composition, antimicrobial, and antioxidant potential of Cymbopogon martinii (Roxb.) Wats. essential oil
Source: Front Plant Sci. 2025 Sep 12;16:1660363. doi: 10.3389/fpls.2025.1660363 (PMC12463950; doi:10.3389/fpls.2025.1660363)
Supplement: Supplementary file 1 [file Table1.docx]

**Influence of growth stage on the chemical composition, antimicrobial, and antioxidant potential of *Cymbopogon martinii* (Roxb.) Wats. essential oil**

**Priyankaraj Sonigra, Mukesh Meena***

Laboratory of Phytopathology and Microbial Biotechnology, Department of Botany, Mohanlal Sukhadia University, Udaipur, Rajasthan, India

***Corresponding author**

Dr. Mukesh Meena

Department of Botany

Mohanlal Sukhadia University, Udaipur, Rajasthan, India

Email: mukeshmeenamlsu@gmail.com / drmukeshmeena321@mlsu.ac.in

**Supplementary Table S1.** Eigenvalues obtained from the correlation matrix through principal component analysis (PCA) of the chemical constituents present in essential oils of *Cymbopogon martinii* during vegetative, reproductive, and post-reproductive growth phases.

| **S. No.** | **Principle Component** | **Eigenvalue** | **Percentage of Variance** | **Cumulative** |
| --- | --- | --- | --- | --- |
| 1. | PC 1 | 42.61196 | 56.07% | 56.07% |
| 2. | PC 2 | 33.38804 | 43.93% | 100.00% |
| 3. | PC 3 | 0 | 0.00% | 100.00% |

**Supplementary Table S2.** Principal component loadings values for major chemical components identified in the essential oils of *Cymbopogon martinii* during vegetative, reproductive, and post-reproductive growth stages.

| **S. No.** | **Variables** | **Principle Component** | | |
| --- | --- | --- | --- | --- |
|  |  | **PC 1** | **PC 2** | **PC 3** |
| 1. | Norbornane | 0.83723 | 0.71907 | 0.75966 |
| 2. | Styrene | -1.02678 | -0.3494 | -0.20941 |
| 3. | Camphene | -0.57939 | -0.96195 | -0.05582 |
| 4. | trans-Decalin | 0.78859 | -0.77811 | 0.00496 |
| 5. | β-Myrcene | -1.07455 | -0.09935 | -0.14903 |
| 6. | p-Cymene | 0.68011 | -0.88518 | -0.27098 |
| 7. | o-Cymene | -0.55531 | -0.9777 | -0.22231 |
| 8. | D-Limonene | -0.89106 | -0.64269 | -0.00194 |
| 9. | D-Sylvestrene | 0.83723 | 0.71907 | -0.13878 |
| 10. | 1,3,8-p-Menthatriene | 1.07864 | -0.00571 | -0.45046 |
| 11. | cis-Linalool oxide | -1.00761 | 0.40705 | 0.08092 |
| 12. | Limonene oxide | 0.32629 | 1.08701 | -0.07547 |
| 13. | (E,E)-Cosmene | 0.83723 | 0.71907 | -0.0618 |
| 14. | 2,6-Dimethyl-1,3,5,7-octatetraene, E,E- | -1.00761 | 0.40705 | 0.08092 |
| 15. | cis-p-Mentha-2,8-dien-1-ol | -0.17038 | 1.12612 | 0.0205 |
| 16. | 4-Pentenylbenzene | -1.00761 | 0.40705 | 0.14691 |
| 17. | Isopinocarveol | -0.10382 | 1.13514 | -0.29949 |
| 18. | β-Cyclocitral | -0.53779 | 0.98859 | 0.05733 |
| 19. | Ethanone, 1-(3-methylphenyl)- | -1.00761 | 0.40705 | -0.00156 |
| 20. | 4-Isopropenylcyclohexanone | -1.00761 | 0.40705 | 0.14691 |
| 21. | trans-Verbenol | 0.385 | 1.06532 | -0.20114 |
| 22. | Myrtenal | -0.99946 | -0.42892 | 0.06951 |
| 23. | trans-p-Mentha-1(7),8-dien-2-ol | 0.58464 | 0.95839 | -0.34596 |
| 24. | Myrtenol | 0.1709 | 1.12603 | 0.05694 |
| 25. | cis-Piperitol | -1.00607 | 0.41129 | -0.0341 |
| 26. | Pulegone | 0.83723 | 0.71907 | -0.0618 |
| 27. | Carveol | 0.33262 | -1.08486 | -0.15125 |
| 28. | 1,6-Dihydrocarveol | -0.02174 | 1.1402 | 0.03768 |
| 29. | trans-Piperitol | 0.85902 | -0.68973 | -0.02925 |
| 30. | trans-Isopiperitenol | -0.6192 | 0.93382 | -0.02595 |
| 31. | cis-Verbenone | -0.3686 | 1.07178 | -0.04557 |
| 32. | cis-Dihydrocarvone | 1.04413 | -0.28623 | -0.04592 |
| 33. | 3-Cyclohexene-1-acetaldehyde, α,4-dimethyl- | -1.00761 | 0.40705 | 0.14691 |
| 34. | Cyclohexene, 4-isopropenyl-1-methoxymethoxymethyl- | -0.2408 | 1.11165 | -0.00758 |
| 35. | 2-Cyclohexen-1-ol, 2-methyl-5-(1-methylethenyl)-, cis- | -1.00761 | 0.40705 | -0.00156 |
| 36. | cis-p-Mentha-1(7),8-dien-2-ol | 1.07832 | 0.02834 | -0.04542 |
| 37. | Carvomenthenal | 0.83723 | 0.71907 | -0.13878 |
| 38. | Piperitone | 0.48755 | -1.01729 | -0.09917 |
| 39. | L-Perillaldehyde | -1.00456 | 0.41539 | -0.11614 |
| 40. | trans-Carveyl acetate | 0.17038 | -1.12612 | 0.00974 |
| 41. | Carvone | -0.53704 | -0.98904 | 0.365 |
| 42. | 5,7-Dodecadiyn-1,12-diol | -1.00761 | 0.40705 | 0.05893 |
| 43. | (S)-Perillyl alcohol | -0.83723 | -0.71907 | 0.13878 |
| 44. | Oxycymol | 0.83723 | 0.71907 | -0.0618 |
| 45. | α-Methylcinnamaldehyde | 0.17038 | -1.12612 | -0.0205 |
| 46. | Bicyclo[3.1.1]hept-3-ene-spiro-2,4'-(1',3'-dioxane), 7,7-dimethyl- | -1.00761 | 0.40705 | 0.14691 |
| 47. | Eugenol | 0.07298 | 1.13782 | 0.14283 |
| 48. | Isoledene | -1.0692 | 0.15067 | 0.05892 |
| 49. | Ageratriol | -0.4789 | 1.02188 | 0.05296 |
| 50. | Isoelemicin | 1.07829 | 0.02984 | -0.15525 |
| 51. | Humulenol-II | 0.83723 | 0.71907 | -0.13878 |
| 52. | Methyl 3,5-tetradecadiynoate | 0.83723 | 0.71907 | -0.13878 |
| 53. | Bergamotol, Z-a-trans- | -0.17038 | 1.12612 | -0.03655 |
| 54. | Methyl 7,9-octadecadiynoate | 0.17038 | -1.12612 | 0.00974 |
| 55. | Methyl 8,10-octadecadiynoate | -0.90948 | 0.61316 | -0.06714 |
| 56. | Methyl 4,6-tetradecadiynoate | -0.94101 | -0.55745 | -0.20769 |
| 57. | 2,5-Octadecadiynoic acid, methyl ester | -0.33398 | 1.08439 | 0.01922 |
| 58. | 10,13-Octadecadiynoic acid, methyl ester | -1.00761 | 0.40705 | 0.14691 |
| 59. | Methyl linoleate | 0.05476 | -1.13897 | -0.01432 |
| 60. | Eicosapentaenoic acid (EPA) | -0.93546 | 0.5678 | -6.11933E-4 |
| 61. | 6,9,12,15-Docosatetraenoic acid, methyl ester | -1.00761 | 0.40705 | -0.00156 |
| 62. | DPPH Assay (IC_50_) | 0.35342 | 1.07748 | -0.50249 |
| 63. | ABTS Assay (IC_50_) | 0.57379 | 0.9657 | -0.11429 |
| 64. | β-Carotene bleaching Assay (IC_50_) | 0.48842 | 1.01682 | 0.20954 |
| 65. | *Escherichia coli* (MIC) | 1.05026 | 0.25997 | -0.24655 |
| 66. | *Escherichia coli* (MBC) | 1.00123 | 0.42427 | -0.72438 |
| 67. | *Salmonella typhi* (MIC) | 1.01628 | 0.38218 | 0.8165 |
| 68. | *Salmonella typhi* (MBC) | 1.06068 | 0.20733 | 0.77077 |
| 69. | *Staphylococcus aureus* (MIC) | 1.07705 | -0.06223 | 0.00547 |
| 70. | *Staphylococcus aureus* (MBC) | 1.06822 | 0.15829 | -0.07023 |
| 71. | *Alternaria alstroemeriae* (MIC) | 1.06585 | -0.17522 | 0.2904 |
| 72. | *Alternaria alstroemeriae* (MFC) | 1.06221 | -0.19839 | 0.66186 |
| 73. | *Fusarium fujikuroi* (MIC) | 0.60632 | 0.94322 | 0.21897 |
| 74. | *Fusarium fujikuroi* (MFC) | 1.07307 | -0.11586 | 0.36821 |
| 75. | *Colletotrichum queenslandicum* (MIC) | 1.02229 | 0.36382 | -0.02127 |
| 76. | *Colletotrichum queenslandicum* (MFC) | 0.76325 | -0.80586 | -0.25109 |
