# Supplementary material for: Influence of growth stage on the chemical composition, antimicrobial, and antioxidant potential of Cymbopogon martinii (Roxb.) Wats. essential oil
Source: Front Plant Sci. 2025 Sep 12;16:1660363. doi: 10.3389/fpls.2025.1660363 (PMC12463950; doi:10.3389/fpls.2025.1660363)
Supplement: Supplementary file 2 [file DataSheet1.docx]

**Influence of growth stage on the chemical composition, antimicrobial, and antioxidant potential of *Cymbopogon martinii* (Roxb.) Wats. essential oil**

**Priyankaraj Sonigra, Mukesh Meena***

Laboratory of Phytopathology and Microbial Biotechnology, Department of Botany, Mohanlal Sukhadia University, Udaipur, Rajasthan, India

***Corresponding author**

Dr. Mukesh Meena

Department of Botany

Mohanlal Sukhadia University, Udaipur, Rajasthan, India

Email: mukeshmeenamlsu@gmail.com / drmukeshmeena321@mlsu.ac.in

**Supplementary Figures and Legends**

**Supplementary Figure S1.** Visual comparison of the color of essential oils extracted from *Cymbopogon martinii* at three growth stages: vegetative, reproductive, and post-reproductive.

**Supplementary Figure S2.** Hierarchical clustered heat map of essential oil compound concentrations across different growth stages of *Cymbopogon martinii*. This heat map displays the distribution and relative abundance of essential oil constituents in *Cymbopogon martinii* across three phenological stages: vegetative, reproductive, and post-reproductive. Each cell represents the concentration of a specific compound, with color intensity indicating its relative abundance, as shown by the gradient scale. Hierarchical clustering was conducted using Ward’s linkage method and Euclidean distance to uncover patterns of chemical variation. The resulting clusters reveal distinct groups of compounds that are differentially expressed at various growth stages, emphasizing the dynamic changes in essential oil biosynthesis throughout the plant's development.

**Supplementary Figure S3.** Antibacterial activity of essential oils from *Cymbopogon martinii* at different growth stages (vegetative, reproductive, and post-reproductive) against three bacterial strains: (A) *Escherichia coli*, (B) *Salmonella typhi*, and (C) *Staphylococcus aureus*. The zone of inhibition is represented as follows: (a) negative control, (b) vegetative stage, (c) reproductive stage, (d) post-reproductive stage, and (e) positive control.

**Supplementary Figure S4.** Correlation matrix between essential oil constituents of *Cymbopogon martinii* and their biological activities. This figure shows the Pearson correlation coefficients between major volatile constituents of *Cymbopogon martinii* essential oil (x-axis) and various biological activities (y-axis), including antioxidant assays (DPPH, ABTS, β-carotene bleaching) and antimicrobial effects (MIC, MBC, MFC) against selected bacterial and fungal strains. Dot color represents the direction of correlation (red: positive; blue: negative), while dot size reflects the strength of the correlation (ranging from –1 to +1). This visualization identifies key compounds with strong associations to specific bioactivities, providing insight into the chemical–bioactivity relationships that contribute to the therapeutic potential of *C. martinii* essential oil.

**Supplementary Figure S5.** Antifungal activity of essential oils from *Cymbopogon martinii* at different growth stages (vegetative, reproductive, and post-reproductive) against three fungal strains: (A) *Alternaria alstroemeriae*, (B) *Fusarium fujikuroi*, and (C) *Colletotrichum queenslandicum*. Fungal mycelial growth inhibition is denoted as follows: (a) control, (b) vegetative stage, (c) post-reproductive stage, (d) reproductive stage, and (e) positive control.
